# Micro-nano-bubble ozonation enhanced thiamethoxam mineralization and toxicity alleviation in wastewater

**DOI:** 10.1016/j.eehl.2025.100202

**Published:** 2025-11-25

**Authors:** Xiuwen Li, Yizhou Wu, Manyi Chen, Ting Rui, Peng Shi, Feng Yang, Zepeng Zhang, Min Hu, Feng Zhang, Xiankun Wu, Qing Zhou, Aimin Li

**Affiliations:** a State Key Laboratory of Water Pollution Control and Green Resource Recycling, School of the Environment, Nanjing University, Nanjing 210023, China; b Key Laboratory of Mesoscopic Chemistry of Ministry of Education (MOE), School of Chemistry and Chemical Engineering, Nanjing University, Nanjing 210023, China; c School of Chemical and Environmental Engineering, Yancheng Teachers University, Yancheng 224002, China

**Keywords:** Neonicotinoid insecticides, Micro-nano-bubble ozonation, Thiamethoxam, Wastewater treatment, Toxicity reduction

## Abstract

Neonicotinoid insecticides (NNIs), including thiamethoxam (TMX), clothianidin, and imidacloprid, are widely used in agriculture to control pests. Consequently, they have been frequently detected in wastewater, posing significant ecotoxicological risks. Conventional ozonation is widely applied for NNI removal but is limited by low mineralization efficiency and high effluent toxicity. However, the mechanisms of the performance limitations and increased toxicity remain unclear, hindering the effective application of ozonation in wastewater treatment. This study constructed a novel micro-nano-bubble ozonation (MNB-O_3_) system, which enhanced the degradation rate of TMX, a representative NNI, by 34.7% and the mineralization efficiency by 176.5%, compared to conventional bubble ozonation (CB-O_3_). MNB-O_3_ also significantly reduced both acute toxicity and neurotoxicity in the ozonated effluents, addressing the issue of high toxicity associated with CB-O_3_. Mechanistically, the formation of hydroxyl radicals (·OH) and singlet oxygen (^1^O_2_) increased substantially and was identified as the primary contributor to TMX degradation. Transformation product (TP) analysis revealed that formaldehyde and acetaldehyde were the key contributors to effluent toxicity, both accumulating in the CB-O_3_ effluent. In contrast, MNB-O_3_ achieved significant reductions in formaldehyde (87.4%) and acetaldehyde (34.6%) concentrations, substantially lowering effluent toxicity. Furthermore, a large-scale MNB-O_3_ application demonstrated excellent performance in removing NNIs and reducing toxicity. This study provides valuable insights into the mechanisms underlying toxicity reduction in MNB-O_3_ and highlights its potential for low-carbon wastewater treatment. By addressing the limitations of CB-O_3_ and reducing the NNIs-related environmental risks, MNB-O_3_ represents a promising advancement in the field of wastewater treatment.

## Highlights

• MNB-O_3_ enhanced TMX degradation by 34.7 ​% and mineralization by 176.5 ​%.
• MNB-O_3_ reduced both acute toxicity and neurotoxicity in ozonated effluents.
• Hydroxyl radicals (·OH) and singlet oxygen (^1^O_2_) drive enhanced TMX degradation.
• MNB-O_3_ lowered the formation of formaldehyde and acetaldehyde by 87.4 ​% and 34.6 ​%.
• MNB-O_3_ showed good performances in a large-scale application.

## Graphical abstract

**Figure:**
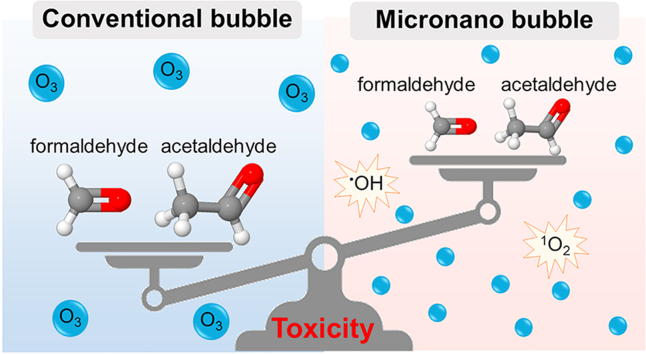

## 1 Introduction

Neonicotinoid insecticides (NNIs) are among the most widely used insecticides globally, owing to their high potency and versatile application methods [1]. Since the 1990s, NNIs have accounted for over 25% of global insecticide sales, contributing approximately $3.7 billion to the market [2]. However, the widespread use of NNIs has led to significant amounts entering wastewater treatment plants (WWTPs) from industrial and urban sources [3]. Due to their poor biodegradability, conventional wastewater treatment processes are inefficient, with over 75% NNIs discharged into surface waters [4]. As neurotoxic compounds, NNIs pose substantial risks to aquatic ecosystems, even at low concentrations [5]. In addition, NNIs and their transformation products (TPs) in WWTPs effluents exacerbate these risks [6], necessitating the development of effective treatment technologies.

Among advanced oxidation processes (AOPs), ozonation is considered cleaner and more energy-efficient compared to methods like Fenton oxidation and electrochemical treatment. It has become a widely adopted technology for advanced wastewater treatment [7], showing promise in removing NNIs such as imidacloprid, acetamiprid, and thiacloprid [[8], [9]]. However, conventional ozonation suffers from limitations, including ozone's low solubility in water, instability, selectivity, and a low mineralization rate [[10], [11], [12]]. Furthermore, treating NNIs with conventional ozonation can increase effluent toxicity, posing risks to receiving water bodies [[13], [14]]. This increased toxicity is likely linked to the formation of highly toxic intermediates during the oxidation process, though the underlying mechanisms remain unclear [15]. Therefore, there is an urgent need to understand the causes of this increased toxicity and to optimize the ozonation treatment process for NNI-contaminated wastewater [16].

Micro-nano-bubbles ozonation (MNB-O_3_), which generates bubbles with diameters smaller than 100 ​μm, has recently gained attention for wastewater treatment due to its unique physicochemical properties [17]. Compared to conventional bubble ozonation (CB-O_3_), MNB-O_3_ enhances ozone utilization, increases ozone concentration at the gas-liquid interface, and improves the removal of organic pollutants from wastewater [18]. Additionally, MNB-O_3_ promotes ozone self-decomposition, generating reactive oxidative species (ROS) such as hydroxyl radical (·OH), singlet oxygen (^1^O_2_), and superoxide radical (O_2_^·−^) [[19], [20]]. These properties suggest that MNB-O_3_ may be particularly effective for NNIs, which share similar nitrogen-containing heterocyclic structures with ortho-positioned nitrogen that are more susceptible to mineralization by ROS [21]. Despite its potential, no studies have yet investigated the application of MNB-O_3_ in NNI-contaminated wastewater treatment, and the performance of MNB-O_3_ for NNI mineralization rate and toxicity reduction in wastewater remains to be examined.

To address the need for advanced treatment and toxicity reduction of NNI-contaminated wastewater, this study constructed an MNB-O_3_ versus CB-O_3_ apparatus and optimized the experimental parameters for MNB-O_3_. Thiamethoxam (TMX), a widely detected NNI in urban water systems [22], was chosen as the target compound. This study evaluated and compared the removal efficiency, mineralization rate, and toxicity reduction of TMX wastewater using MNB-O_3_ and CB-O_3_. Degradation and toxicity reduction mechanisms were explored through scavenger experiments, electron paramagnetic resonance (EPR) analysis, TPs identification, and density functional theory (DFT) calculations. Furthermore, the performance and energy demand of the MNB-O_3_ process were assessed in a large-scale application. The findings aim to provide valuable scientific insights into the use of MNB-O_3_ for the effective treatment of NNI-contaminated wastewater.

## 2 Materials and methods

### 2.1 Chemicals

TMX was procured from Alta Scientific Co., Ltd. (Tianjin, China). Additional reagents, such as furfuryl alcohol (FFA), para-benzoquinone (pBQ), para-chlorobenzoic acid (pCBA), metronidazole (MDE), 2,2-dimethyl-3,4-dihydro-2H-pyrrole 1-oxide (DMPO), 4-oxo-2,2,6,6-tetramethylpiperidine (TEMP), NaS_2_O_3_, and KI, were supplied by Aladdin Chemistry Co., Ltd. (Shanghai, China). All chemicals had purities exceeding 98%. Chromatographic-grade methanol (MeOH) was sourced from Sigma Aldrich Inc. (Saint Louis, MO, USA).

### 2.2 Experimental apparatus

The experimental apparatus is depicted in Fig. S1. The reaction column featured an outer layer with an external circulating-water cooling system to maintain a constant reaction temperature. Ozone was generated using an ozone generator (XM-S, Xinmei, China), with high-purity oxygen (99.999%) as the feed gas. For the MNB-O_3_ system, ozone was introduced into an MNB generator (ZJC-NM-200L, Zhongjing, China), producing ozone micro-nano-bubbles. For the CB-O_3_ system, ozone macro bubbles were generated using a titanium plate at the reactor's base. The bubble diameters for both systems, captured using a high-speed camera (v2640, Phantom, USA), are shown in Fig. S2.

### 2.3 Experimental procedure

The TMX solution was prepared using ultrapure water and secondary sedimentation tank effluent from an industrial wastewater treatment plant. The water quality parameters of the industrial wastewater are listed in Table S1. A 10 ​mg/L TMX solution in ultrapure water was used for TPs identification, while a 50 ​μg/L TMX solution in ultrapure water and wastewater was used for ROS identification and wastewater treatment performance evaluations. In addition, a 10 ​mg/L TMX wastewater solution was tested to verify the removal efficiency under industrial discharge scenarios during the TMX manufacturing process. The ozone gas flow rate was set at 0.5 ​L/min, with an ozone concentration of 60 ​g/m^3^. Samples were collected at regular intervals and immediately treated with 10 ​mM NaS_2_O_3_ to quench the reaction. All samples were filtered through 0.45 ​μm membranes before further analysis. The degradation kinetics of TMX were determined by comparing the reaction rates with and without scavengers, as outlined in Text S1 [19]. Removal kinetics of TMX were fitted using pseudo-first-order kinetics [22], as shown in Eq. 1.

(1) $$\ln\left(C_{t}/C_{0}\right)=-kt$$

where *C*_*t*_ and *C*_0_ are the concentrations of TMX (mg/L or μg/L) at time *t* and the initial time (0), respectively, and *k* (min^−1^) is the observed degradation rate constant.

### 2.4 ROS identification

To determine the exposures of ROS, three probes, pCBA, pBQ (10 ​mg/L each), and MDE (20 ​mg/L) were spiked in the reaction solution of both systems. The concentrations of these three probes were set according to Tang et al. [23] and our pre-experiments. The exposures of ROS were calculated using Eqs. (2), (3), (4) based on the observed removal efficiency of these probes. Detailed information about the analysis methods for these probes is provided in Text S2, and the second-order rate constants of the probes with different ROS are presented in Table S2. In addition, to evaluate the contribution of individual ROS, scavengers were added at a concentration of 10 ​mM: MeOH for ·OH, FFA for ·OH and ^1^O_2_, and pBQ for ·OH, ^1^O_2_, and O_2_^·−^. The calculation method and the second-order rate constants of the scavengers with different ROS are presented in Text S1 and Table S2, respectively. In addition, the radical ·OH and ^1^O_2_ were detected using EPR spectrometry (ESP300E, Bruker, Germany), with DMPO and TEMP at 100 ​mM as trapping agents, respectively.

(2) ln([pCBA]0/[pCBA])=kOH·,pCBA∫[OH·]dt+kO2·−,pCBA∫[O2·−]dt+kO12,pCBA∫[O12]dt

(3) ln([MDE]0/[MDE])=kOH·,MDE∫[OH·]dt+kO2·−,MDE∫[O2·−]dt+kO12,MDE∫[O12]dt

(4) ln([pBQ]0/[pBQ])=kOH·,pBQ∫[OH·]dt+kO2·−,pBQ∫[O2·−]dt+kO12,pBQ∫[O12]dt

where, **∫**[^*·*^*OH*]d*t*, **∫**[*O*_*2*_^*·−*^]d*t*, and **∫**[^*1*^*O*_*2*_]d*t* were ROS exposures. [*probe*] and [*probe*]_0_ were the probe concentrations during reaction and at the initial time, respectively.

### 2.5 NNIs and TPs identification

TPs of aldehydes were derivatized and analyzed using ultra-performance liquid chromatography with ultraviolet detection (UPLC-UV, 1260 Infinity, Agilent, USA) equipped with a C_18_ column (4 ​μm, 4.6 ​mm ​× ​150 ​mm, Agilent). A 20 ​μL sample was injected into the chromatograph at a column temperature of 25 ​°C. The mobile phase was a mixture of acetonitrile, MeOH, and water (V:V:V ​= ​30:20:50) at a flow rate of 1 ​mL/min, and the detection wavelength was 360 ​nm. The details of the derivatization process are described in Text S3. Other TPs and TMX were detected by an ultra-performance liquid chromatography–quadrupole time-of-flight tandem mass spectrometry (UPLC-QTOF-MS/MS, TripleTOF 5600, AB SCIEX, USA) equipped with a C_18_ column (4 ​μm, 4.6 ​mm ​× ​150 ​mm, Agilent). A 10 ​μL sample was injected into the chromatograph at a column temperature of 35 ​°C. The mobile phases were ultra-pure water containing 0.1% formic acid (mobile phase A) and MeOH (mobile phase B) at a flow rate of 1 ​mL/min. The gradient of the mobile phase is presented in Table S3. The samples were analyzed in positive-ion mode, with 55 psi for both gas1 and gas2 and 35 psi for the air-curtain gas. The ion source temperature was set to 550 ​°C, the voltage to +5500 ​V, and the de-clustering voltage to 60 ​V. The concentrations of NNIs in industrial wastewater were determined as described in our previous study [22].

### 2.6 DFT calculation

The TMX molecular structure was constructed using GaussView 6.0 and optimized with the M062X functional and def2TZVP basis sets in Gaussian 16. Frequency analysis was conducted at the same level to ensure that the optimized structure represented a minimum on the potential energy surface. Single-point energy calculations were performed using the M062X/def2TZVP level with a solvation model (SMD) to account for solvent effects. Atomic dipole moment-corrected Hirshfeld (ADCH) charges, frontier molecular orbitals, Fukui functions, and electrostatic potential (ESP) were calculated using Multwfn 3.8 [[24], [25]]. The ESP distribution was visualized with VMD 1.9.3 [26].

### 2.7 Toxicity assessment

Acute toxicities were assessed using aquatic organisms representing three trophic levels: *Vibrio fischeri* (luminescent bacterium), *Daphnia magna* (water flea), and *Danio rerio* (zebrafish embryo). Bioluminescence inhibition of samples was measured after 15 ​min of exposure using the Biotoxicity Analyzer (Microtox® M500, Modern Water, USA). Mortality rates of water flea and zebrafish embryo were recorded after 48 ​h of exposure, following the national standard GB/T 13266–91, and the guideline 236 of the Organization for Economic Co-operation and Development, respectively. The acute toxic unit (TUa) for each organism was calculated using Eq. 5 or Eq. 6, depending on whether the inhibition rate (or mortality rate) of undiluted water samples was below or above 50% [27]. Toxicity classifications are summarized in Table S4.

(5) TUa ​= ​Inhibition rate (or mortality rate)/0.5

(6) TUa ​= ​1/IC_50_ (or EC_50_)

Neurotoxicity was evaluated by analyzing the effects of wastewater samples on the spontaneous movement of zebrafish embryos at 24 ​h post-fertilization (hpf) [28]. Acute toxicity of TMX and its TPs to fish, water flea, and algae was estimated using ECOSAR (v2.0), while developmental toxicity and mutagenicity were assessed with T.E.S.T. (v5.1.1.0), as detailed in our previous study [8]. Chemical structures were visualized using ChemDraw Professional 2016.

### 2.8 Statistical analysis

All tests were conducted in triplicate, with results expressed as the average (AVG) ​± ​standard deviation (SD). Data normality and variance homogeneity were verified using Shapiro–Wilk's test and Levene's test, respectively. Parametric tests were applied for datasets meeting normality and homogeneity assumptions. Statistical comparisons were conducted using one-way analysis of variance (ANOVA) with Dunnett's post-test in GraphPad Prism 8.3. A *p*-value of <0.05 was considered statistically significant.

## 3 Results

### 3.1 MB-O_3_ achieved superior TMX degradation performance than CB-O_3_

**Fig. 1:**
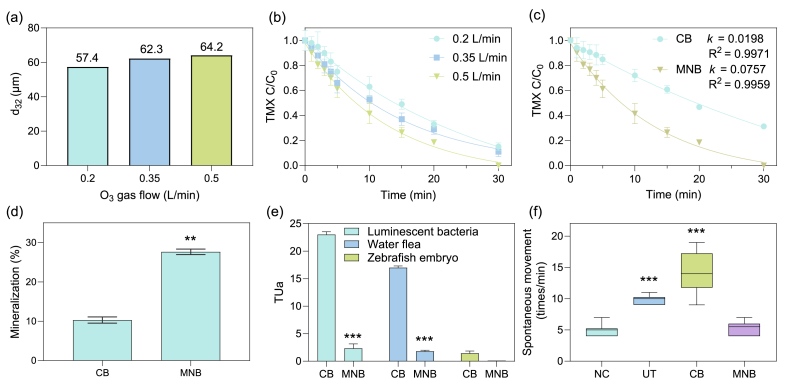
(a) Sauter mean diameter (d_32_) of bubbles under varying O_3_ gas flow rate in the MNB-O_3_ system. (b) TMX degradation under different O_3_ gas flow rates in the MNB-O_3_ system. (c) Dynamics of TMX degradation in the MNB-O_3_ and CB-O_3_ systems. (d) Mineralization rates of MNB-O_3_ and CB-O_3_ after 30 ​min of reaction. (e) Acute toxicity of the effluents treated by MNB-O_3_ and CB-O_3_. (f) Spontaneous movement of zebrafish embryo exposed to effluents from the MNB-O_3_ and CB-O_3_ systems. Experimental conditions: reactor volume ​= ​1.5 ​L, [TMX] ​= ​50 ​μg/L in wastewater, [O_3_ gas flow] ​= ​0.5 ​L/min, [O_3_] ​= ​60 ​g/m^3^, reaction temperature ​= ​25 ​°C, initial pH unadjusted. For panels (d) and (e), significance between MNB-O_3_ and CB-O_3_ is indicated. For panel (f), significance is noted between the treated samples and the NC group. ∗∗*p* ​< ​0.01, ∗∗∗*p* ​< ​0.001. NC, negative control; UT, untreated group.

The Sauter mean bubble diameter (d_32_) increased from 57.4 to 64.2 ​μm as the O_3_ gas flow rate increased from 0.2 to 0.5 ​L/min, with no significant difference observed among the test groups (Fig. 1a). The highest TMX degradation rate was achieved at a flow rate of 0.5 ​L/min (Fig. 1b). Beyond 0.5 ​L/min, the MNB generator became unstable. Therefore, a gas flow rate of 0.5 ​L/min was maintained for both MNB-O_3_ and CB-O_3_ systems in subsequent experiments. As shown in Fig. 1c, the CB-O_3_ system achieved 68.7% TMX removal after 30 ​min of reaction, whereas the MNB-O_3_ system reached 100% TMX removal within the same time frame. The degradation rate constant for TMX in the MNB-O_3_ system (0.0757 ​min^−1^) was 3.8 times higher than that in the CB-O_3_ system (0.0198 ​min^−1^). At an initial TMX concentration of 10 ​mg/L, MNB-O_3_ also achieved 100% removal after 45 ​min of ozonation, whereas CB-O_3_ removed only 48.3% (Fig. S3). Additionally, mineralization rates of the two systems were assessed in a real industrial wastewater matrix (Fig. 1d). After 30 ​min of oxidation, CB-O_3_ achieved a mineralization rate of only 10.3%, whereas MNB-O_3_ achieved a significantly higher mineralization rate of 27.6%, representing a 168% improvement over CB-O_3_.

The acute toxicity of the treated effluents was evaluated (Fig. 1e). For CB-O_3_-treated effluent, the TUa values were 23.0 (high acute toxicity) for luminescent bacteria, 17.0 (high acute toxicity) for water flea, and 1.50 (acute toxicity) for zebrafish. In contrast, MNB-O_3_-treated effluent exhibited significantly lower acute toxicity, with TUa values below 10 (acute toxicity) for luminescent bacteria and water flea, and 0.1 (no acute toxicity) for zebrafish. On average, MNB-O_3_ reduced effluent acute toxicity by 89.8% compared to CB-O_3_. In addition to acute toxicity, neurotoxicity assessments were conducted, considering the neurotoxic effects of TMX (Fig. 1f). The mean spontaneous movement of zebrafish embryos in the negative control (NC) group was 5 times/min, while the untreated (UT) group exhibited a significantly higher movement rate of 10 times/min. Following CB-O_3_ treatment, the spontaneous movement further increased to 14 times/min, significantly exceeding the NC group's rate (*p* ​< ​0.001). In contrast, MNB-O_3_ treatment restored zebrafish embryos' spontaneous movement to a normal level of 5 times/min, consistent with the NC group.

### 3.2 MB-O_3_ enhanced the exposures and contributions of ·OH and ^1^O_2_ in TMX degradation

**Fig. 2:**
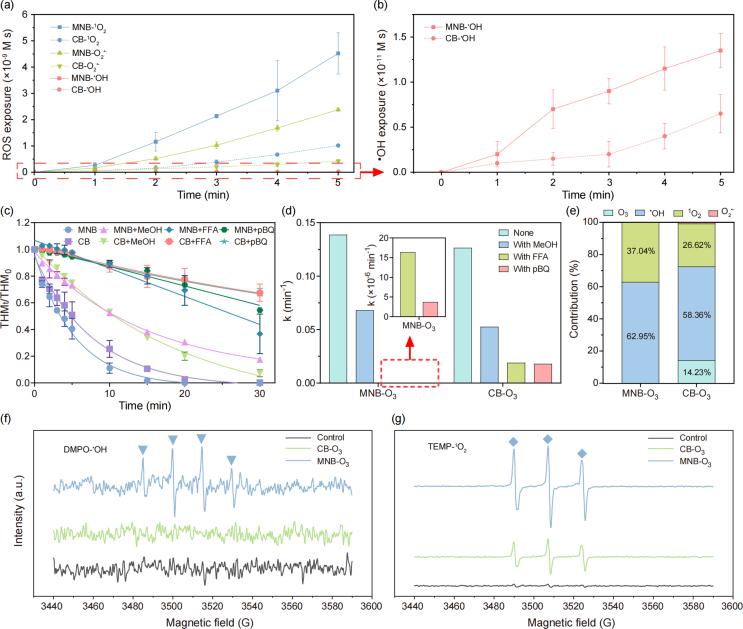
(a) Evolution of ·OH, ^1^O_2_, and O_2_^·−^ exposures; (b) evolution of ·OH exposures; (c) degradation curves of TMX in the presence and absence of scavengers; (d) kinetic rate constants for TMX degradation; (e) contribution of ROS to TMX removal; (f) EPR spectra for DMPO-·OH; (g) EPR spectra for TEMP-^1^O_2_. Same experimental conditions as in Fig. 1.

ROS exposure was calculated in 5 ​min, because all spiked pBQ was removed totally within 5 ​min in the MNB-O_3_ system. As shown in Fig. 2a and b, the exposure of ·OH, ^1^O_2_, and O_2_^·−^ in the MNB-O_3_ system were 2.1, 4.4, and 5.6 folds of that in the CB-O_3_ system. In comparison with ^1^O_2_ and O_2_^·−^, ·OH exposure was much lower in both systems. Contributions of different ROS during TMX degradation were further assessed by adding ROS scavengers. TMX removal in both ozonation processes was significantly inhibited upon the addition of various ROS scavengers (Fig. 2c). The kinetic rate constants for TMX removal without scavengers were 0.1265 ​min^−1^ for CB-O_3_ and 0.1387 ​min^−1^ for MNB-O_3_ (Fig. 2d). Upon the introduction of scavengers of MeOH, FFA, and pBQ, the kinetic rate constants decreased. In CB-O_3_ process, the contributions were determined to be 14.23% (O_3_), 58.36% (·OH), 26.62% (^1^O_2_) and 0.79% (O_2_^·−^) (Fig. 2e). Comparatively, in the MNB-O_3_ process, the contributions of O_3_ and O_2_^·−^ were nearly negligible, with TMX removal primarily driven by ·OH (62.95%) and ^1^O_2_ (37.04%). Although the ·OH exposure was lower than other ROS, it was still the main contributor to TMX degradation. Conversely, even O_2_^·−^ exposure reached 2.4 ​× ​10^−8^ in the MNB-O_3_ system, its contribution to TMX degradation was marginal. To further confirm the presence of ·OH and ^1^O_2_ in both ozonation processes, their levels were semi-quantitatively assessed using EPR. Signals corresponding to DMPO-·OH and TEMP-^1^O_2_ in MNB-O_3_ were notably stronger than those observed in CB-O_3_, indicating a higher generation of these ROS in MNB-O_3_ (Fig. 2f and g).

### 3.3 Reaction sites of TMX proposed by DFT calculation

**Fig. 3:**
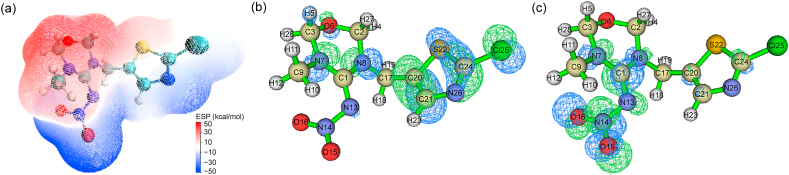
(a) ESP distribution map of the TMX molecule, with red regions indicating positive charges and blue regions indicating negative charges. Isosurface maps of the HOMO (b) and LUMO (c) for the TMX molecule, with green and blue regions representing positive and negative phases of the wave function, respectively.

The preferential reaction sites of TMX during the ozonation process were predicted based on DFT calculations. The optimized geometric structure of the TMX molecule is shown in Fig. S3. Based on the ESP distribution on the van der Waals surface of the TMX molecule, the thiazole ring, –NO_2_ moiety on the oxadiazine ring, and the –CH_2_– moiety connecting the thiazole and oxadiazine rings were identified as falling within the blue region (Fig. 3a), suggesting that these sites are most susceptible to attack by electrophilic reagents [28]. Additionally, the ADCH charges of the TMX molecule are presented in Table S5. Atoms with more negative charge, including 6(O) (−0.283932), 13(N) (−0.392805), 15(O) (−0.465028), 16(O) (−0.426129), 26(N) (−0.314138), were identified as particularly vulnerable to electrophilic attack [28]. Analysis of the molecular orbitals revealed that the highest occupied molecular orbital (HOMO) was at orbit 75, with an energy of −0.30198 a.u., and the lowest unoccupied molecular orbital (LUMO) was at orbit 76, with an energy of −0.025567 a.u. The HOMO-LUMO energy gap was 0.276413 a.u., indicating the chemical stability of TMX [20]. As shown in Fig. 3b and c, the orbital components of the HOMO were mainly located in the thiazole and oxadiazine rings, indicating these regions were most prone to electrophilic attack, consistent with the ESP distribution results. Meanwhile, the LUMO orbital components were concentrated in the oxadiazine ring and its –NO_2_ moiety, suggesting that these sites are most vulnerable to nucleophilic reactions. To further elucidate the reaction activity of individual atoms in the TMX molecule, Fukui functions and double descriptors were calculated, and the results are presented in Table S6 and Fig. S4. The Δf values of atoms 17(C), 20(C), 21(C), 22(S), 24(C), and 26(N) were negative, indicating their susceptibility to attack by electrophilic reagents [29]. In addition, atoms 20(C) and 21(C) exhibited relatively higher values of f^0^, highlighting their vulnerability to free radical attack [30]. In summary, DFT calculations suggest that TMX oxidation preferentially occurs on the thiazole and oxadiazine rings. Specifically, atoms 6(O), 13(N), 15(O), 16(O), 26(N), 17(C), 20(C), 21(C), 22 (S), and 24(C) are the most likely sites for electrophilic attack, while 20(C) and 21(C) are the primary targets for ·OH.

### 3.4 Degradation pathway of TMX proposed by TPs identification

Based on reaction sites analysis and mass spectrum data, a total of 8 ​TPs were identified during the ozonation of TMX Table S7). These TPs were detected in both CB-O_3_ and MNB-O_3_ processes, suggesting that bubble size did not influence the types of TPs formed. Structures of TP1–TP5 were proposed by combining mass spectra interpretation and DFT calculations. For example, the molecular formula of TP1 (*m/z* 250) was determined to be C_6_H_8_ClN_5_O_2_S using the Formula Finder function in the Peak View 1.3 software (AB SCIEX). Compared to TMX (molecular formula C_8_H_10_ClN_5_O_3_S), TP1 exhibited two fewer carbon atoms, two fewer hydrogen atoms, and one fewer oxygen atom. According to DFT calculations, oxidation of TMX preferentially occurs at the oxadiazine ring. Hydrogen abstraction at the 4(H) and 5(H) sites of the oxadiazine ring could lead to the breakage of C = N bonds on both sides, resulting in the formation of TP1. Secondary mass spectra of TP1 further revealed fragmentation peaks at *m/z* 175 and 133 (Fig. S6). The peak at *m/z* 175 [M ​+ ​H−46−29]^+^ likely resulted from two cleavages: (1) N–N bond cleavage in the –N–NO_2_ moiety (NO_2_ loss), and (2) C–N bond cleavage in the –C–NH–CH_3_ moiety (NH = CH_2_ loss). Subsequent cleavage of the C–N bond connected to the thiazole ring could yield the fragment at *m/z* 133 [M ​+ ​H−46−29−42]^+^, validating the structural formula of TP1. Similar reasoning approaches were employed to deduce the structures of TP2–TP5, as detailed in Text S4.

**Fig. 4:**
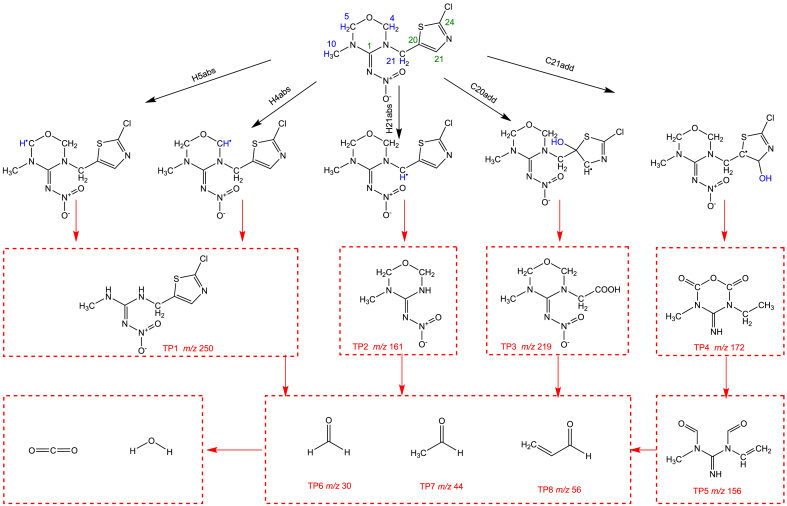
Degradation pathways of TMX during CB-O_3_ and MNB-O_3_ processes.

Apart from TP1–TP5 detected by UPLC-QTOF-MS/MS, TP6 (formaldehyde), TP7 (acetaldehyde), and TP8 (acrolein) were identified using UPLC-UV. In summary, TP1–TP8 were generated in both the CB-O_3_ and MNB-O_3_ processes. The proposed degradation pathway is depicted in Fig. 4. After the hydrogen abstraction reactions of 4(H) and 5(H), the C–N bond on the oxadiazine ring was broken to produce TP1. After the hydrogen abstraction reaction of 21(H), the C–N bond connecting 17(C) to the oxadiazine ring was broken to produce TP2. 20(C) and 21(C) underwent a free radical addition reaction, and the C–S and C–C bonds on the thiazole ring were broken to generate TP3 and TP4. TP4 was further oxidized, and the C–O bond on the original oxadiazine ring was broken to generate TP5. When TP1–TP5 were further oxidized, the aldehydes TP6–TP8 were generated, and were finally mineralized to generate CO_2_ and H_2_O.

### 3.5 MNB-O_3_ was effective in reducing the formation of high-toxic TPs

To elucidate the mechanisms underlying the toxicity reduction achieved by MNB-O_3_, the toxicity of TMX and its TPs was evaluated based on the identified TPs (Table S8). Toxicity endpoints included acute toxicity (to fish, water flea, and algae), developmental toxicity, mutagenicity, and bioaccumulation factor. The results showed that TMX exhibited relatively low acute toxicity to fish, but was harmful to both water flea and algae. TMX was also identified as developmentally toxic and mutagenic. Several TPs showed reduced acute toxicity compared to TMX, although they remained developmentally toxic and mutagenic. Specifically, the developmental toxicity values of TP4 and TP5 were 1.25 and 1.1, respectively, exceeding that of the parent compound TMX (1.04). Among these aldehydes TPs (TP6–TP8), TP6 (formaldehyde) had an LC_50_ value of 5.87 ​mg/L for green algae, categorizing it as “toxic”. TP7 (acetaldehyde) was considered “harmful” to all three tested organisms. TP8 (acrolein) exhibited a high level of toxicity, with an LC_50_ value of 0.12 ​mg/L for fish, categorizing it as “very toxic”.

To further investigate the relationship between TPs and overall effluent toxicity, the acute toxicity, neurotoxicity, and concentration of each TP were analyzed under CB-O_3_ and MNB-O_3_ (Fig. S11). Due to the lack of standards for TP1 to TP5, their concentrations could not be precisely quantified; instead, peak intensities were used as proxies for their relative levels [15]. Correlation analysis was then performed to examine the relationship between the concentrations of TPs and toxicity endpoints, including luminescence inhibition and spontaneous movement, which were proven to be sensitive in *Section* 3.1 (Fig. 1e and f). Among all TPs, TP6 (formaldehyde) exhibited the highest correlation coefficients with luminescence inhibition (R^2^ ​= ​0.98) and spontaneous movement (R^2^ ​= ​0.96), followed by TP7 (acetaldehyde), with correlation coefficients of 0.76 and 0.79, respectively (Fig. S12). Compared to CB-O_3_, the MNB-O_3_ process significantly reduced the concentrations of TP6 (formaldehyde) and TP7 (acetaldehyde) by 87.44% and 34.56%, respectively (Fig. S11), demonstrating its effectiveness in mitigating the formation of highly toxic TPs.

### 3.6 MNB-O_3_ showed promising performance in a large-scale application

**Fig. 5:**
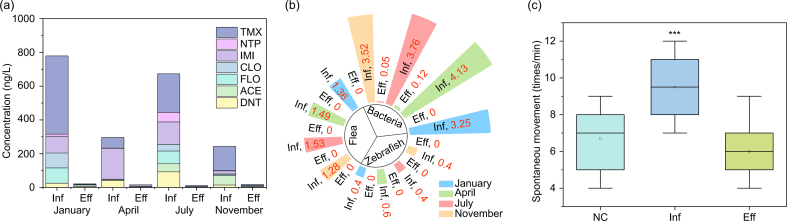
Performance of the MNB-O_3_ system in an industrial application. (a) Concentrations of NNIs in the influent and effluent of the MNB-O_3_ system, including nitenpyram (NTP), imidacloprid (IMI), clothianidin (CLO), flonicamid (FLO), acetamiprid (ACE), and dinotefuran (DNT). (b) TUa values for luminescent bacteria, water fleas, and zebrafish embryos before and after MNB-O_3_ treatment. (c) Spontaneous movements of the zebrafish embryos at 24 hpf exposed to influent (Inf), effluent (Eff), and the negative control (NC). Asterisks (∗∗∗) indicate significant differences between groups at *p* ​< ​0.001.

To evaluate the practicality of the MNB-O_3_ in real-world engineering, this system was implemented as an advanced treatment step in an industrial WWTP in Jiangxi Province, China, with an actual treatment load of 3000 ​t per day (Fig. S13). This WWTP receives wastewater containing NNIs produced by pesticide manufacturers. The performance of MNB-O_3_ was monitored across four seasons, and the water quality before and after MNB-O_3_ treatment is presented in Table S9. Seven kinds of NNIs were detected in the influent of the MNB-O_3_ treatment stage, with concentrations ranging from 243.6 to 779.4 ​ng/L. Among them, TMX was the most prevalent insecticide (63.3–463.4 ​ng/L). After MNB-O_3_ treatment, the NNI concentrations in the effluent were reduced to a range of 11.8–21.2 ​ng/L, achieving removal efficiencies of 92.6%–98.2% (Fig. 5a). The TUa value of the influent was 3.25–4.13, 1.28–1.53, and 0.4–0.6 for luminescent bacteria, water flea, and zebrafish, respectively. After MNB-O_3_ treatment, the acute toxicity for these three organisms dropped to nearly zero (Fig. 5b). In terms of neurotoxicity, the influent caused zebrafish embryos to exhibit a spontaneous movement frequency of 10 times/min, significantly higher than the NC group (7 times/min). Encouragingly, after MNB-O_3_ treatment, the spontaneous movement frequency decreased to a normal level of 6 times/min (Fig. 5c). The overall cost of this WWTP was calculated as 7.61 yuan/t, while the MNB-O_3_ (excluding electricity) stage accounted for 0.90 yuan/t, according to the design manual (Text. S5). The energy consumption of the MNB-O_3_ system was also assessed according to the power of electrical equipment (Table S10), and the total energy consumption per ton of wastewater was calculated to be 0.83 ​kWh (0.50 yuan/t). In this case, the cost of MNB-O_3_ process was 1.40 yuan/t, accounting for 18.4% of the overall cost.

## 4 Discussion

Clean water and sanitation (SDG 6) is a key United Nations Sustainable Development Goal, where effective wastewater treatment plays a pivotal role in protecting the ecological environment and achieving this target. In China, pollution prevention and control strategies have focused on addressing long-term issues such as water pollution [31]. As scientific understanding advances, the focus of wastewater treatment has shifted from solely removing pollutants to a broader emphasis on environmental risk management [32]. This paradigm shift is especially crucial for industrial wastewater containing toxic chemicals, where mitigating effluent toxicity becomes more critical than mere pollutant reduction. In this study, we applied the MNB-O_3_ technology, which showed remarkable effectiveness in treating NNI-contaminated wastewater at both low (μg/L) and high (mg/L) initial concentrations. Such results demonstrated that MNB-O_3_ was not only efficient in the advanced treatment of mixed wastewater, but also suitable for the treatment of manufacturing wastewater of TMX factories. More importantly, it significantly mitigated the effluent toxicity in laboratory tests and a large-scale application of industrial wastewater treatment, largely securing the safety of the finished wastewater.

The observed toxicity reduction with MNB-O_3_ can be attributed to its enhanced capability to decompose and mineralize NNIs, thus curbing the formation of highly toxic TPs such as TP6 (formaldehyde) and TP7 (acetaldehyde). Formaldehyde is carcinogenic and mutagenic, classified as a class I carcinogen by the International Agency for Research on Cancer [33]. It can affect glial and neuronal cells in the brain, causing neurotoxicity [34]. Acetaldehyde impairs mitochondrial function and leads to brain tissue damage and cognitive dysfunction [35]. These short-chain aldehydes are often formed during conventional ozonation processes [[36], [37]] and other AOPs, such as ultraviolet-driven AOPs [38] and electrocoagulation [39]. While strategies such as catalytic ozonation and biological post-treatment have been developed to address this issue, these methods often require additional chemical inputs or extended treatment processes, driving up both capital and operational costs [[40], [41]]. In contrast, MNB-O_3_ demonstrated a more efficient and cost-effective solution by significantly reducing the concentrations of these aldehydes without necessitating additional investments.

Besides its economic and energy-saving benefits, MNB-O_3_ also showed other advantages. For example, in comparison with conventional ozonation, the system only requires an MNB generator, making it more convenient for transforming the original treatment process. This simplicity is especially valuable because ozonation is the most widely used AOP in industrial WWTPs. In addition, it is also easy to operate and maintain through an automatic control system. Recently, other AOPs, such as Fenton or Fenton-like, UV, and catalytic ozonation, were also reported to be efficient in removing NNIs [42]. However, these technologies have some drawbacks in the application of real wastewater treatment. For example, Fenton needs acidic reaction conditions [43] and input of H_2_O_2_, which is dangerous to transport and store. Besides, it will generate ion sludge during the reaction, requiring additional treatment [44]. UV is energy-consuming, and UV254 lamps have a limited lifespan (usually several thousand hours) and need to be replaced regularly [45]. Catalytic ozone oxidation often faces problems such as catalyst deactivation and treatment in practical applications, which can cause secondary pollution. In contrast, MNB-O_3_ is clean and can react in an ambient environment, showing great potential for large-scale promotion.

The superior performance of MNB-O_3_ in enhancing the mineralization of NNIs is closely linked to the generation of ROS within the system. Two key factors may contribute to this: (1) the accumulation of OH^−^ ions at the interfaces of MNBs, which enhances ozone self-decomposition and promotes ROS generation [46], and (2) the localized chemical potential gradients dispersion caused by the collapse of MNBs, which produces additional ROS [47]. Among these ROS, the predominance of ·OH in TMX degradation across both CB-O_3_ and MNB-O_3_ is unsurprising, given its status as the strongest oxidizer with no selectivity towards organic pollutants [48]. Direct O_3_ oxidation in the CB-O_3_ system played a minor role in degrading TMX, primarily due to the electron-withdrawing moieties (−Cl, −S, and −NO_2_) that deactivate the primary attack sites for O_3_ on NNIs, such as C=C and C=N bonds [15]. Other studies also evidenced that direct oxidation by O_3_ was insufficient for removing NNIs, and that ·OH was the main contributor, overlooking other ROS like ^1^O_2_ [[12], [14]]. However, in this study, ^1^O_2_ also played a critical role in NNI degradation during MNB-O_3_ treatment, likely due to its high reactivity towards electron-rich sites on TMX [49]. Similar findings have been reported in previous studies, such as the degradation of oxytetracycline by ^1^O_2_ in the MNB-O_3_ system [23]. Furthermore, DFT calculations suggest that sites on NNIs, including specific oxygen, nitrogen, carbon, and sulfur atoms, are vulnerable to electrophilic attack by ·OH and ^1^O_2_, facilitating efficient mineralization of TMX and its TPs [50].

It is worth noting that, although similar kinds of TPs were generated in both systems, the concentrations of TPs, as well as the exposure and contributions of different ROS, changed when the ozone bubble decreased from millimeter scale to micrometer scale, indicating MNB may further alter the pathways and reaction mechanisms of TMX degradation [51]. The exposure of the major ROS (·OH and ^1^O_2_) increased by several folds in the MNB-O_3_ system. Among them, ^1^O_2_ was proved to be efficient in removing aldehydes [50], while ·OH was inefficient [52]. Based on the above results, we proposed that MNB-O_3_ promoted the generation of ·OH and ^1^O_2_, and subsequently promoted the decomposition of TMX and formation of aldehydes. In the meantime, ^1^O_2_ played an important role in eliminating aldehydes, securing safety of the effluent. However, in CB-O_3_ system, although most TMX was decomposed, the amount of ^1^O_2_ was inadequate to remove the accumulated aldehydes, resulting in the high toxicity of the effluent.

As a primary engineering measure for water pollution prevention, WWTPs collect and purify wastewater, thus mitigating their environmental impact [53]. In the large-scale application (3000 ​t/d) analyzed in this study, MNB-O_3_ achieved NNI removal efficiencies ranging from 92.6% to 98.2%, demonstrating stable performance on NNI removal and toxicity reduction. The removal efficiencies were significantly higher than those of other industrial WWTPs as reported in previous investigations [22], but lower than the laboratory values (100%), which may be affected by the suspended solids in the real wastewater matrix [54]. Cost analysis is also crucial for evaluating the feasibility of wastewater treatment technologies. In this study, the energy consumption of MNB-O_3_ was measured at 0.83 ​kWh/t. Due to the absence of a direct comparison between MNB-O_3_ and CB-O_3_ in the same project, energy consumption for CB-O_3_ was obtained from published literature (1.7–2.5 ​kWh/t), significantly higher than that of MNB-O_3_ [55]. This energy efficiency is critical, as ozone generation accounts for 50%–75% of the total costs in traditional ozonation processes [17]. Although the incorporation of an MNB generator increased initial energy input, the improved ozone utilization and accelerated pollutant degradation reduced hydrological residence time and ozone dosage, offsetting these additional costs [56]. Thus, MNB-O_3_ offers a more sustainable and economically viable solution for wastewater treatment.

## 5 Conclusion

The findings of this study highlight the significant advantages of MNB-O_3_ in degrading NNIs and reducing effluent toxicity. By minimizing the formation of aldehyde TPs, MNB-O_3_ outperforms other modified ozonation technologies that require additional catalysts or prolonged processes. Furthermore, the high reactivity and stability of ^1^O_2_ in saline environments suggest that MNB-O_3_ could be particularly effective for treating wastewater with high salinity, broadening its applicability [57]. In the context of global carbon-neutrality and energy-conservation goals, energy-efficient wastewater treatment technologies are becoming increasingly important. MNB-O_3_'s low energy consumption and seamless integration into existing wastewater treatment infrastructures make it an attractive choice for industrial applications. This study not only demonstrates the efficacy of MNB-O_3_ in treating NNI-contaminated wastewater but also provides insights into its underlying toxicity reduction mechanisms, further reinforcing its potential for widespread adoption.

Despite these advancements, challenges remain. The performance of MNB-O_3_ in varying wastewater matrices, particularly those with complex compositions or high levels of suspended solids, requires further investigation. Future research should focus on optimizing operational parameters, such as bubble size and residence time, to enhance its adaptability for diverse wastewater types. Additionally, long-term assessments of MNB-O_3_'s environmental impact and cost-effectiveness in real-world applications are essential to ensure its sustainable deployment. In conclusion, MNB-O_3_ represents a promising advancement in AOPs, offering an effective, energy-efficient, and scalable solution for wastewater treatment. By addressing both pollutant removal and effluent toxicity, it aligns with the evolving goals of wastewater treatment and environmental risk management. Future studies and technological refinements will undoubtedly expand its potential, paving the way for more sustainable wastewater treatment practices.

## CRediT authorship contribution statement

**Feng Yang:** Investigation. **Peng Shi:** Writing – review & editing, Supervision, Resources, Funding acquisition. **Min Hu:** Investigation. **Zepeng Zhang:** Investigation. **Yizhou Wu:** Visualization, Software. **Xiuwen Li:** Writing – original draft, Methodology, Investigation, Data curation, Conceptualization. **Ting Rui:** Visualization, Software. **Manyi Chen:** Investigation, Formal analysis. **Xiankun Wu:** Conceptualization. **Feng Zhang:** Writing – review & editing. **Aimin Li:** Project administration, Funding acquisition. **Qing Zhou:** Writing – review & editing

## Declaration of competing interests

The authors declare no conflicts of interest.
