# Supplementary material for: Micro-nano-bubble ozonation enhanced thiamethoxam mineralization and toxicity alleviation in wastewater
Source: Eco Environ Health. 2025 Nov 25;4(4):100202. doi: 10.1016/j.eehl.2025.100202 (PMC12723015; doi:10.1016/j.eehl.2025.100202)
Supplement: Multimedia component 1 [file mmc1.docx]

**Supporting Information for**

**Micronano-bubble ozonation enhanced thiamethoxam mineralization and toxicity alleviation in wastewater**

Xiuwen Li^a^, Yizhou Wu^a^, Manyi Chen^a^, Ting Rui ^a^, Peng Shi^a*^, Feng Yang^a^, Zepeng Zhang^a^, Min Hu^a^, Feng Zhang^b^, Xiankun Wu^c^, Qing Zhou^a^, Aimin Li^a*^

^a^ State Key Laboratory of Water Pollution Control and Green Resource Recycling, School of the Environment, Nanjing University, Nanjing 210023, China.

^b^ Key Laboratory of Mesoscopic Chemistry of Ministry of Education (MOE), School of Chemistry and Chemical Engineering, Nanjing University, Nanjing 210023, China

^c^ School of Chemical and Environmental Engineering, Yancheng Teachers University, Yancheng 224002, China.

***Corresponding author.**

**Email:** shipeng@nju.edu.cn (P. Shi)

# Text S1. Calculation of kinetic constants and contributions of ^•^OH, ^1^O_2_ and O_2_^•−^ to THM removal.

MeOH is considered as a scavenger for ^•^OH, and FFA considered as a scavenger for both ^•^OH and ^1^O_2_, while pBQ is considered as a scavenger for ^•^OH, ^1^O_2_ and O_2_^•−^. The calculation of kinetic constants of THM removal by ^•^OH, ^1^O_2_ and O_2_^•−^ was as follows:

$k_{O_{3}}=k_{pBQ}$ （1）

$k_{\bullet OH}=k_{THM}-k_{MeOH}$ （2）

$k_{{1O}_{2}}=k_{MeOH}-k_{FFA}$ （3）

$k_{O_{2}^{\bullet-}}=k_{FFA}-k_{pBQ}$ （4）

The calculation of the relative contributions of ^•^OH, ^1^O_2_ and O_2_^•−^ to THM removal was as follows:

$f_{O_{3}}=\frac{k_{pBQ}}{k_{THM}}\times100\%$ （5）

$f_{\bullet OH}=\frac{k_{THM}-k_{MeOH}}{k_{THM}}\times100\%$ （6）

$f_{{1O}_{2}}=\frac{k_{MeOH}-k_{FFA}}{k_{THM}}\times100\%$ （7）

$f_{O_{2}^{\bullet-}}=\frac{k_{FFA}-k_{pBQ}}{k_{THM}}\times100\%$ （8）

Where, *k*_SMX_ is first-order kinetic constant of THM removal without scavengers, *k*_MeOH_ is first-order kinetic constant of THM removal with MeOH, *k*_FFA_ is first-order kinetic constant of THM removal with FFA, *k*_pBQ_ is first-order kinetic constant of THM removal with pBQ.

# Text S2. Analysis methods for pCBA and pBQ and MDE.

The concentrations of pCBA and pBQ and MDE were all analyzed using ultra-performance liquid chromatography with ultraviolet detection (UPLC-UV, 1260 Infinity, Agilent, USA) equipped with a C_18_ column (4 μm, 4.6 mm × 150 mm, Agilent). 20 μL of sample was injected into the chromatograph at column temperature of 25 °C. The mobile phase for pCBA was a mixture of MeOH and 0.1% formic acid solution (V/V = 55/45) at a flow rate of 1 mL/min, and the detection wavelength was 234. The mobile phase for pBQ and MDE was a mixture of acetonitrile and 0.1% formic acid solution (V/V = 15/85) at a flow rate of 1 mL/min, and the detection wavelength was 245 and 319, respectively.

# Text S3. Derivatization of aldehyde TPs.

TPs of aldehydes were detected after derivatization: 0.4 mL of sodium citrate buffer (0.8 M citric acid + 0.2 M sodium citrate) and 0.3 mL of 2,4-DNPH were mixed with 10 mL of water samples before pH adjusting to 3 with HCl or NaOH. Then samples were placed in a water bath at 40 ± 1 °C for 1 h. Subsequently, aldehydes were extracted three times with 2 mL of dichloromethane after the addition of 0.15 g of NaCl, and the lower layer of solution was collected and evaporated to dryness under a gentle nitrogen stream at 35 °C and reconstituted with 0.5 mL of MeOH.

# Text S4. Reasoning process for TP2–TP5.

The major ionic fragment mass of TP2 was 161, and Formular Finder speculates that its molecular formula may be C_4_H_8_N_4_O_3_. Comparing with THM, TP2 lost 4 C atoms, 2 H atoms, 1 Cl atom, 1 N atom and 1 S atom. According to the results of DFT calculations, 17(C) between the thiazole and oxadiazine rings was vulnerable to the attacked by electrophilic reaction. Thus, when the C–N between 17(C) and 8(N) was broken, TP2 can be obtained as shown in Table S6. In addition, one peak with an *m/z* of 42 fragments can be found through the secondary mass spectra of TP2 (Fig. S6). This may be formed by the breakage of the N–N below TP2, which removes –NO_2_, and the simultaneous breakage of the C–N on the oxadiazine ring, which removes –CH_3_–N–CH_2_–O–CH_2_ and forms 42[M+H−46−73]^+^, thus verifying the structural formula of TP2.

The major ionic fragment mass of TP3 was 219, and Formular Finder speculates that its molecular formula may be C_6_H_8_N_4_O_5_. Comparing with THM, TP3 lost 2 C atoms, 2 H atoms, 1 Cl atom, 1 N atom, and 1 S atom less, and 1 O atom more. According to DFT calculations, 21(C) might be attacked by ^•^OH, while 20(C) and 22(S) was suitable for electrophilic attack. As a result, the C–C and C–S bonds were broken, and formed TP3. In addition, one peak with an *m/z* of 98 fragments can be found through the secondary mass spectrometry of TP3. This could be 98[M+H−46−75]^+^, or 98[M+H−46−75]^+^ and 98[M+H−29−46−46]^+^, as shown in Fig. S7.

The major ionic fragment mass of TP4 was 172, and Formular Finder speculates that its molecular formula may be C_6_H_9_N_3_O_3_. TP4 lost 2 C atoms, 1 H atom, 1 Cl atom, 2 N atoms, and 1 S atom compared with THM. As illustrated by DFT analysis, the –NO_2_ moiety on the oxadiazine ring might be attacked by both electrophilic reagents and nucleophilic reaction, causing the N–N bond broken, and leading to the formation of TP4. The secondary mass spectrometry of TP4 were presented in Fig. S8, which validate the structural formula of TP4.

TP5 has a major ionic fragment mass of 156, and Formular Finder speculates that its molecular formula may be C_6_H_9_N_3_O_2_. comparing to TP4, TP5 had one less O atom. This may be formed by a breakage of the C–O bonds on the ring structuure. In addition, one peak with an *m/z* of 98 fragments can be found by the secondary mass spectra of TP5 (Fig. S9). This may be formed by the 98[M+H−58]^+^ formed by the C–N on the left side of TP5 undergoing fracture and shedding –CH_2_–NH–CO, thus verifying the structural formula of TP5.

# Text S5. Overall cost analysis according to the design manual.

The process flow chart of this industrial WWTP was presented in Fig. S14. The designed treatment load was 7000 t/d, despite the actual operating load was 3000 t/d during our research. So, the overall cost was calculated according to the designed treatment load:

(1) Electricity Cost

The WWTP’s daily wastewater treatment capacity is 7000 t/d. The theoretical daily electricity consumption is approximately 24794.76 kWh, with an electricity price of 0.8 yuan/kWh. Using a power factor of 0.75:

E1 = 24794.76 × 0.75 × 0.8 / 7000 = 2.13 yuan/t

(2) Chemical and Material Costs

The chemicals and consumables used in this WWTP mainly include phosphorus removal agents, PAM (anionic), PAC, liquid oxygen, catalysts consumed for ozone generation, nutrient solutions required for terminal denitrification, etc.

The average daily consumption of liquid oxygen is 6.24 tons, priced at approximately 1000 yuan/t.

Phosphorus removal agent is dosed at 0.05% of the water volume. The daily consumption is 3.5 tons, priced at 700 yuan/t.

PAC is dosed at 0.008% of the water volume. The average daily consumption is 0.56 tons, priced at 2400 yuan/t.

PAM is dosed at 0.0015% of the water volume. The average daily consumption is 0.105 tons, priced at approximately 23000 yuan/t.

Denitrification nutrient solution uses industrial sodium acetate, dosed at 0.015% of the water volume. The average daily consumption is 1.05 tons, priced at approximately 1000 yuan/t.

Activated carbon is replaced every two years, with each replacement amounting to 96 tons. Waste activated carbon is treated as solid waste, with disposal costs calculated at 2600 yuan/ton. Additional activated carbon is replenished at 5000 yuan/ton.

Total chemical and material costs:

E2 = (6.24 × 1000 + 3.5 × 700 + 0.56 × 2400 + 0.105 × 23000 + 1.05 × 1000) / 7000 + 96 × (5000 + 2600) / (7000 × 365 × 2) = 2.07 yuan/t

(3) Labor Costs

The WWTP employs 16 workers, with wages calculated at 200 yuan/day per person.

E3 = 200 × 16 / 7000 = 0.46 yuan/t

(5) Routine Maintenance Costs

Calculated at 1.2 million yuan/year:

E4 = 1200000 / (365 × 7000) = 0.47 yuan/t

(6) Sludge Disposal Costs

The plant generates 0.83 t/d of biological sludge and 5.86 t/d of physicochemical sludge. Sludge disposal costs are calculated at 2600 yuan/t.

E5 = 2600 × (0.83 + 5.86) / 7000 = 2.48 yuan/t

In summary, the total water treatment cost for the plant is:

E_total = E1 + E2 + E3 + E4 + E5 = 7.61 yuan/t

Among these, the treatment cost for MNB-O_3_ (excluding electricity) primarily consists of liquid oxygen:

E_ MNB-O_3_ = 6.24 × 1000 / 7000 = 0.90 yuan/t, accounting for 11.83% of the total cost.


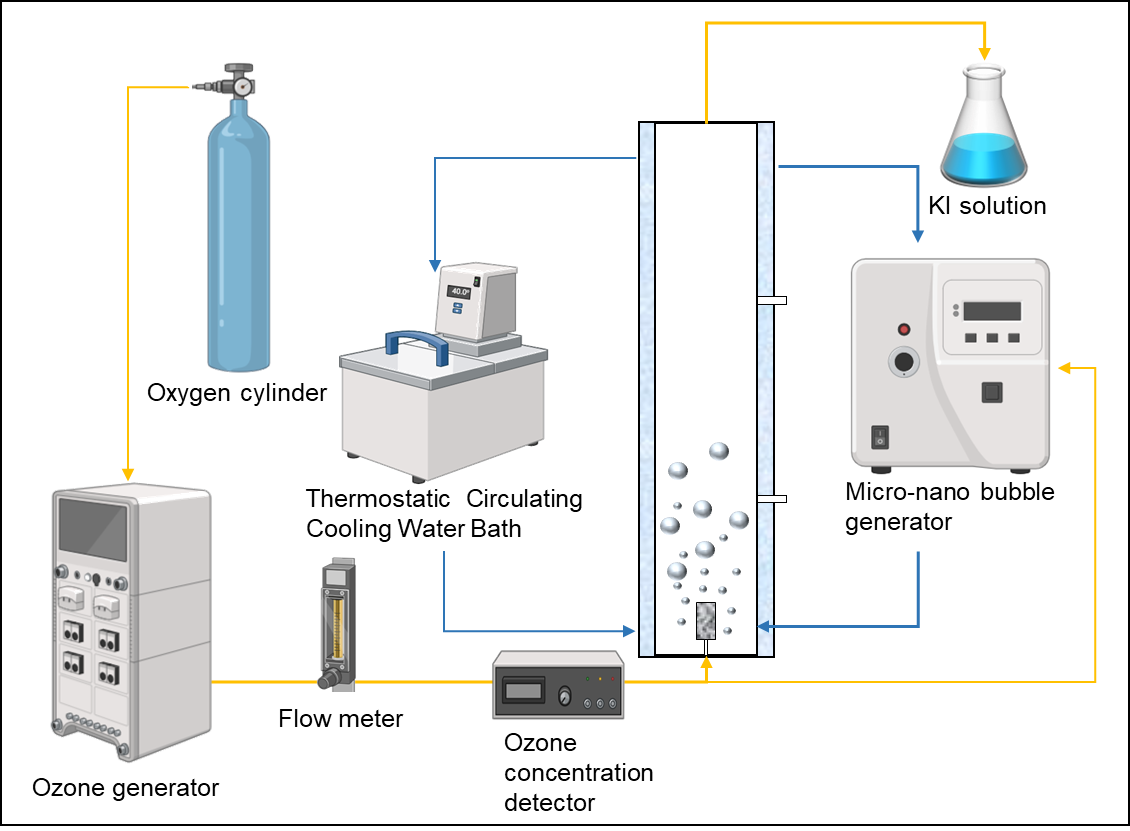


# Fig. S1. Experimental apparatus


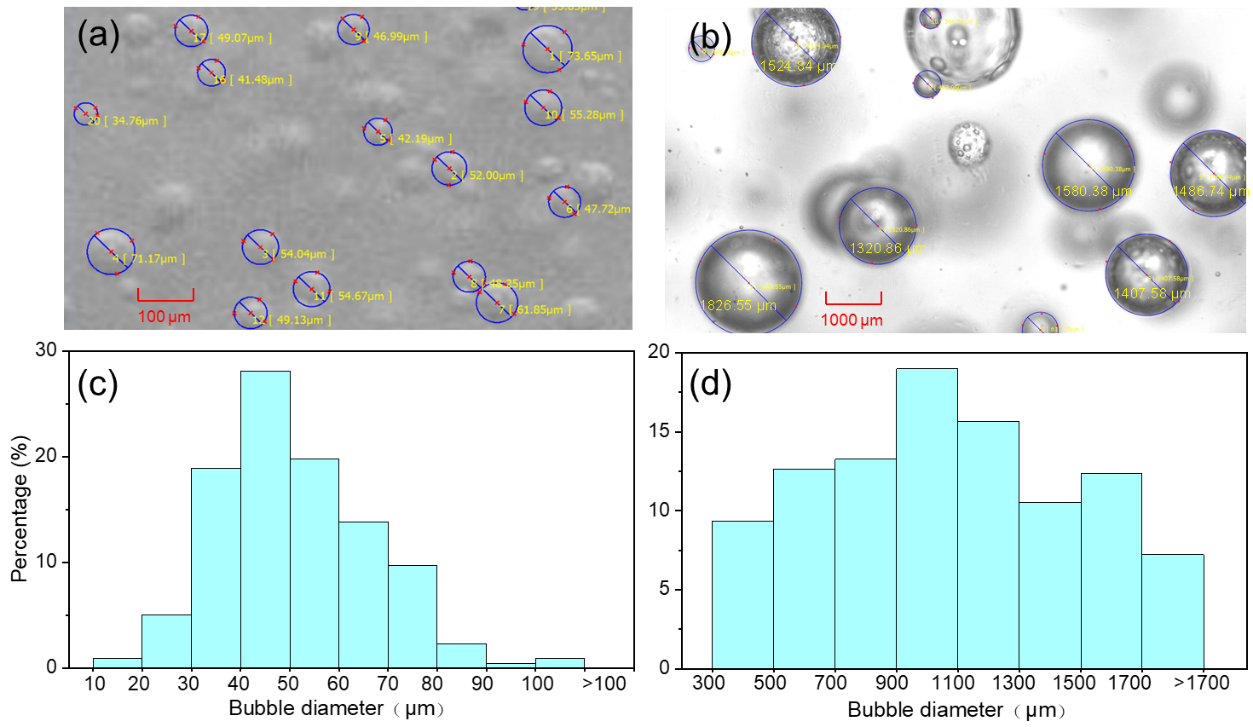


# Fig. S2. Pictures and size distribution of ozone bubbles. (a) and (c) were in MNB-O_3_ system, while (b) and (d) were in CB-O_3_ system. The photo was taken by a high-speed camera.





# Fig. S3. Removal efficiency of TMX. Experimental conditions: reactor volume = 1.5 L, [TMX] = 10 mg/L in wastewater, [O_3_ gas flow] = 0.5 L/min, [O_3_] = 60 g/m^3^, [reaction temperature] = 25 °C, initial pH unadjusted.


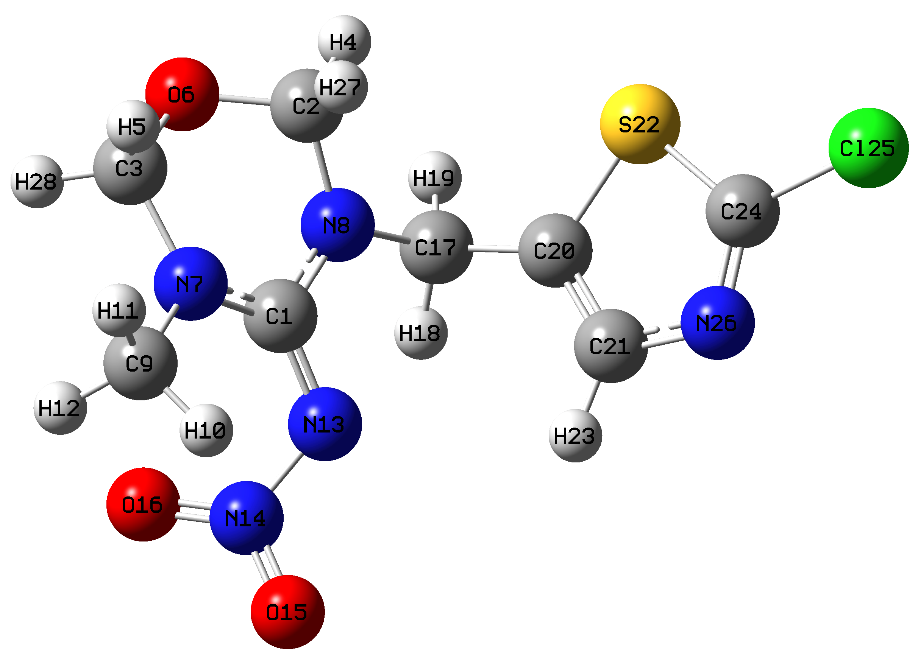


# Fig. S4. Optimized geometric structure of the THM molecule.


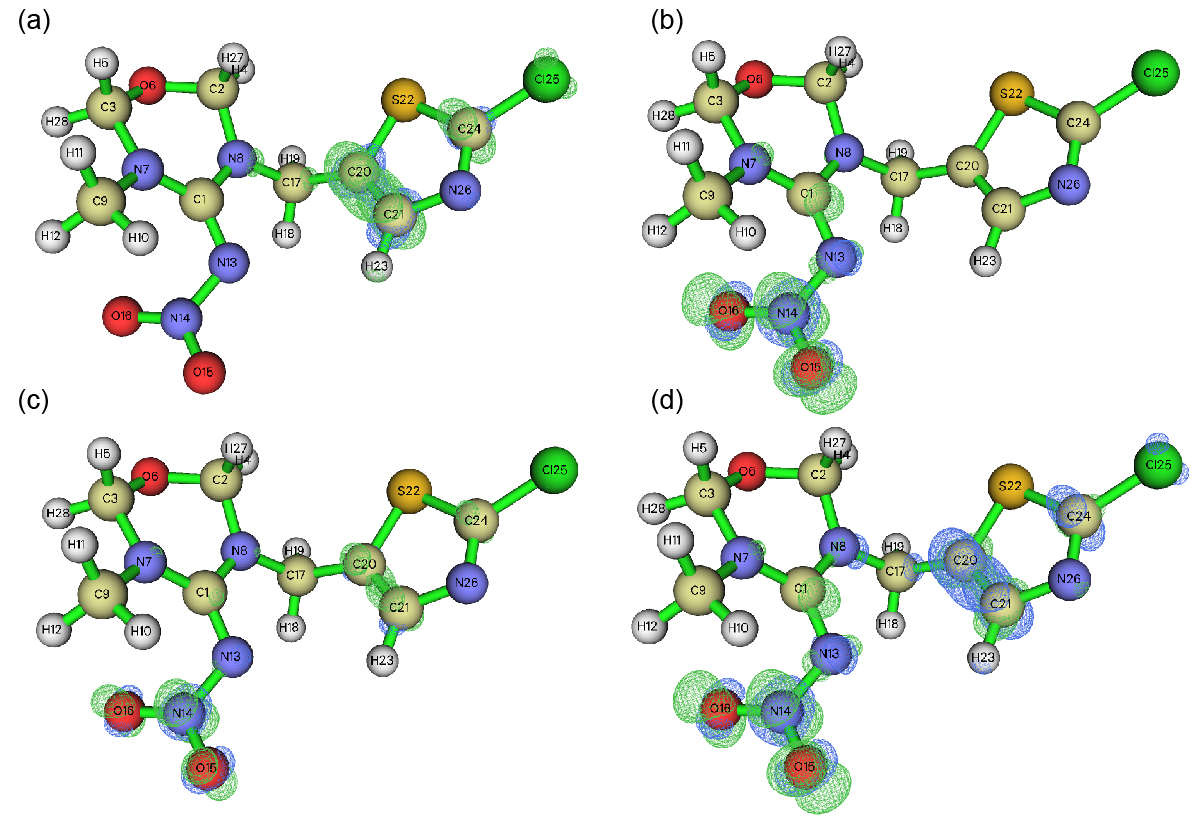


# Fig. S5. Isosurface maps of Fukui functions and double descriptor: (a) f^−^(r); (b) f^+^(r); (c) f^0^(r); (d) Δf(r). Green color for positive region and blue color for negative region.


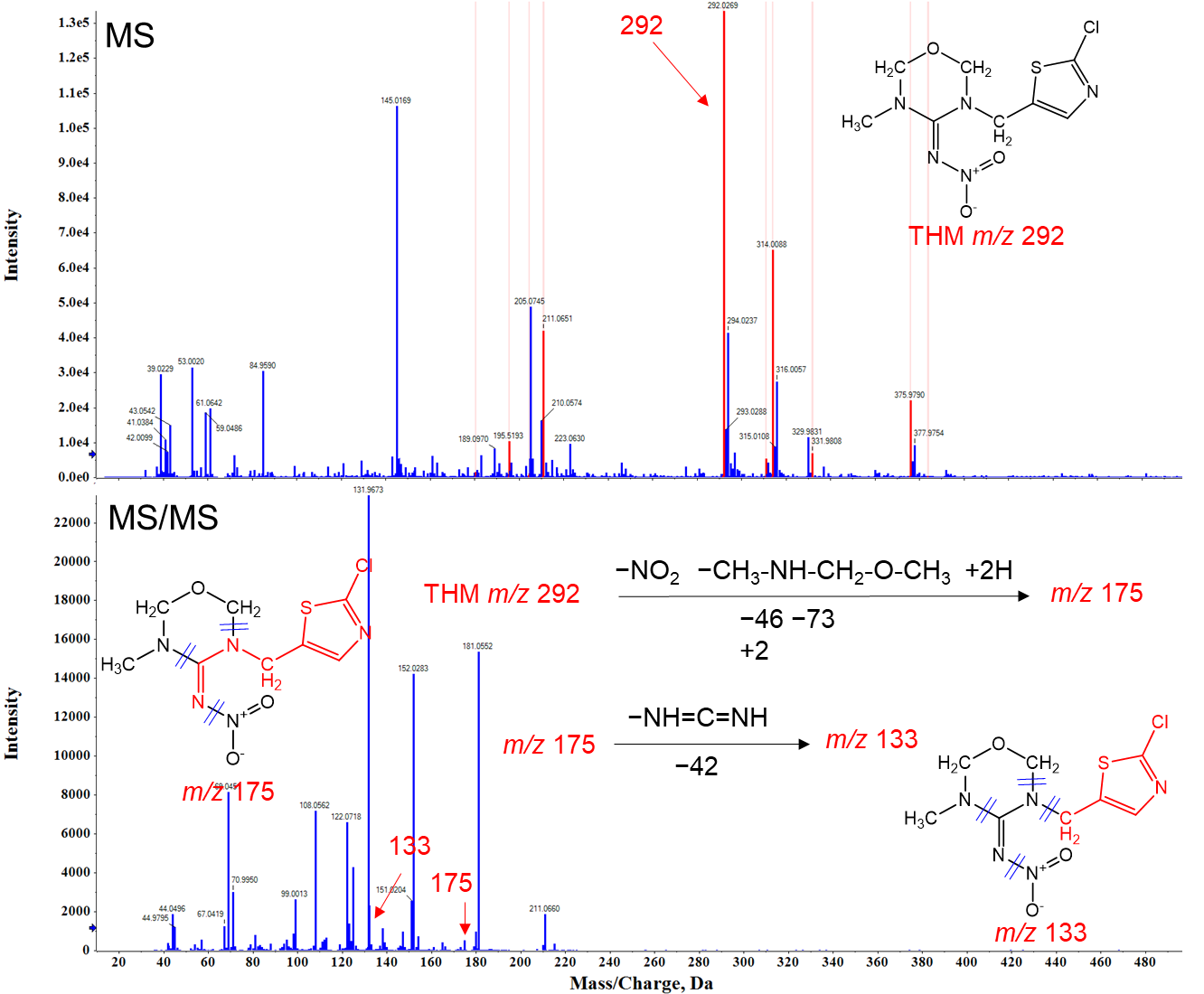


# Fig. S6. QTOF mass spectrums of THM.


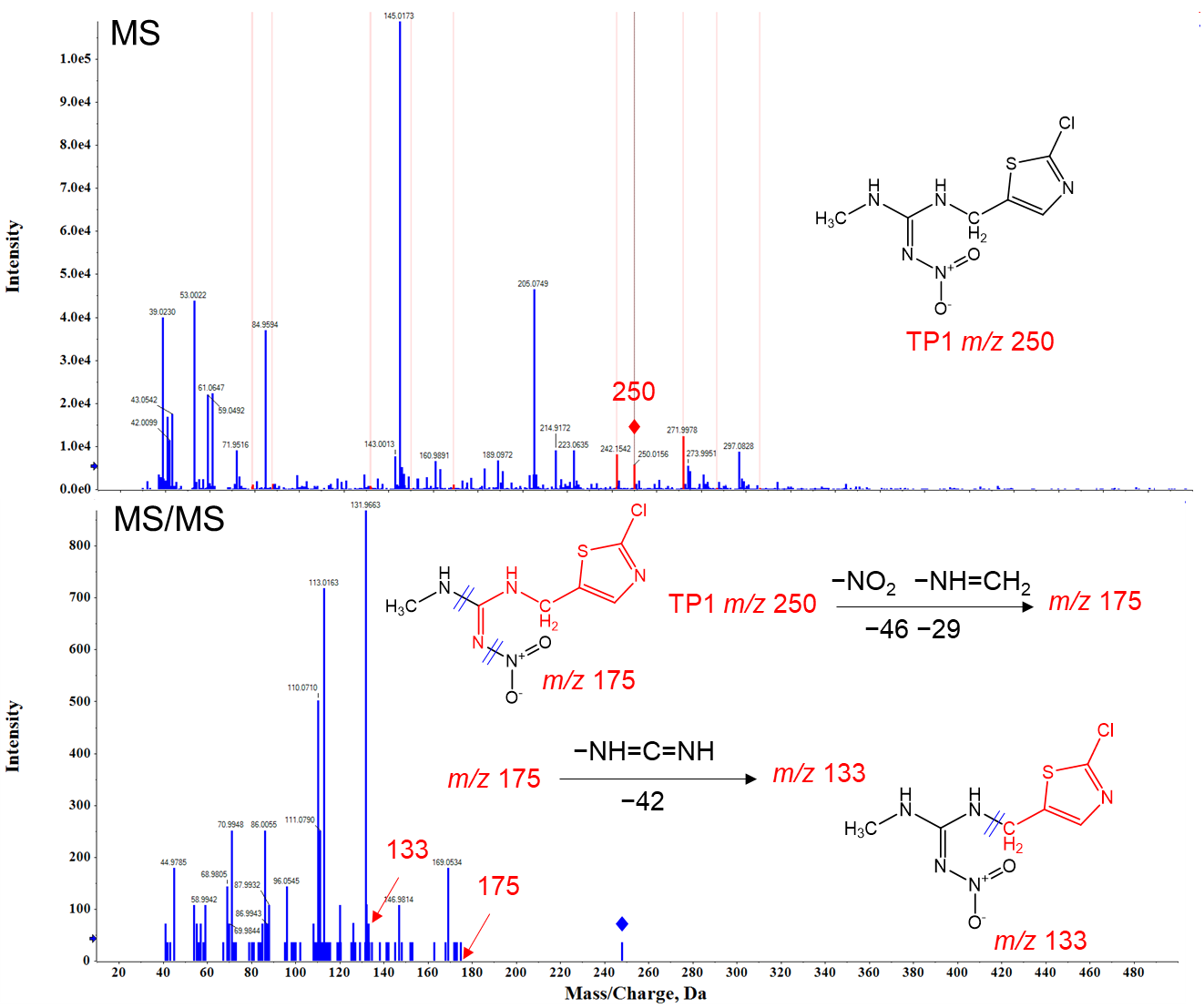


# Fig. S7. QTOF mass spectrums of TP1.


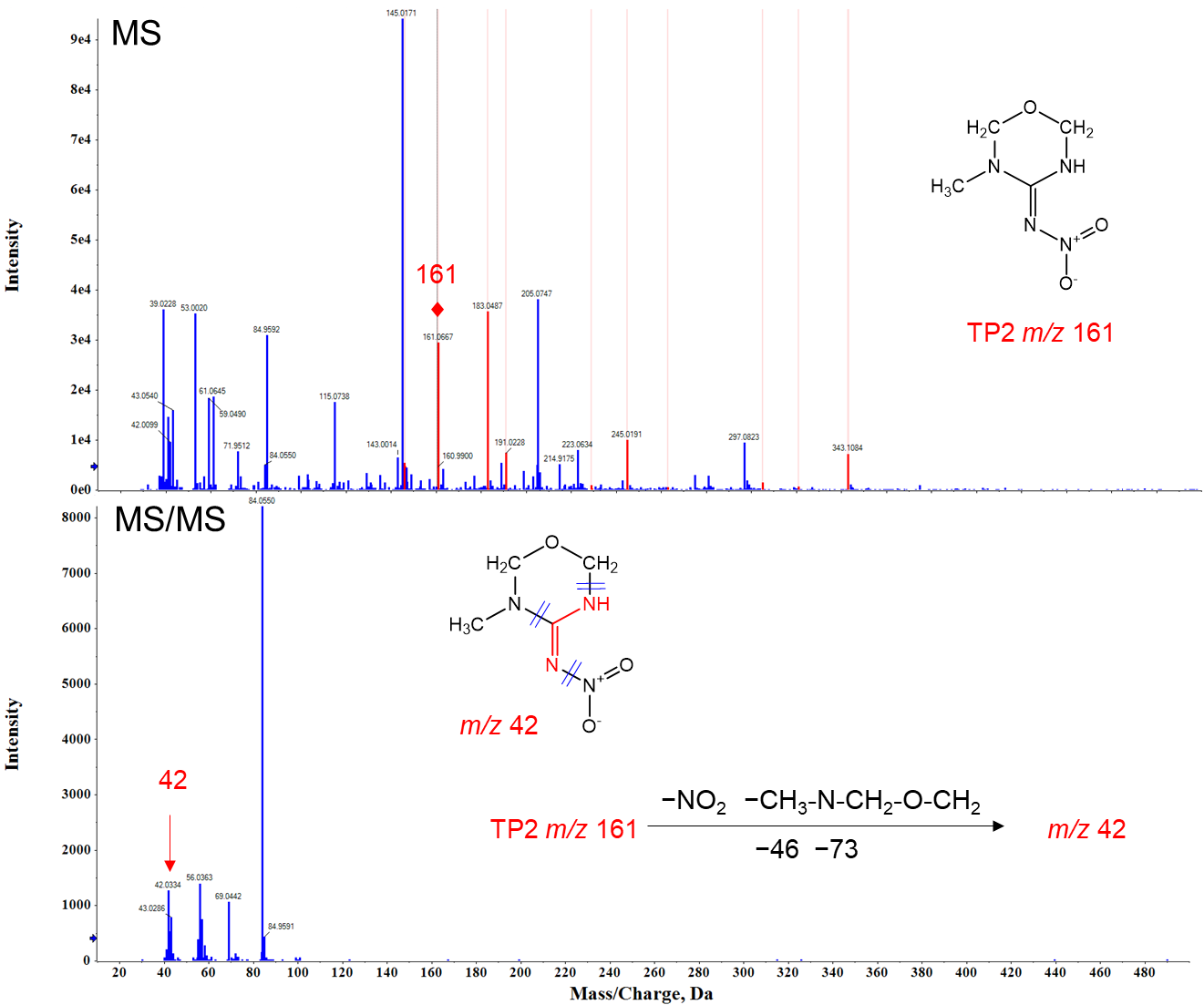


# Fig. S8. QTOF mass spectrums of TP2.


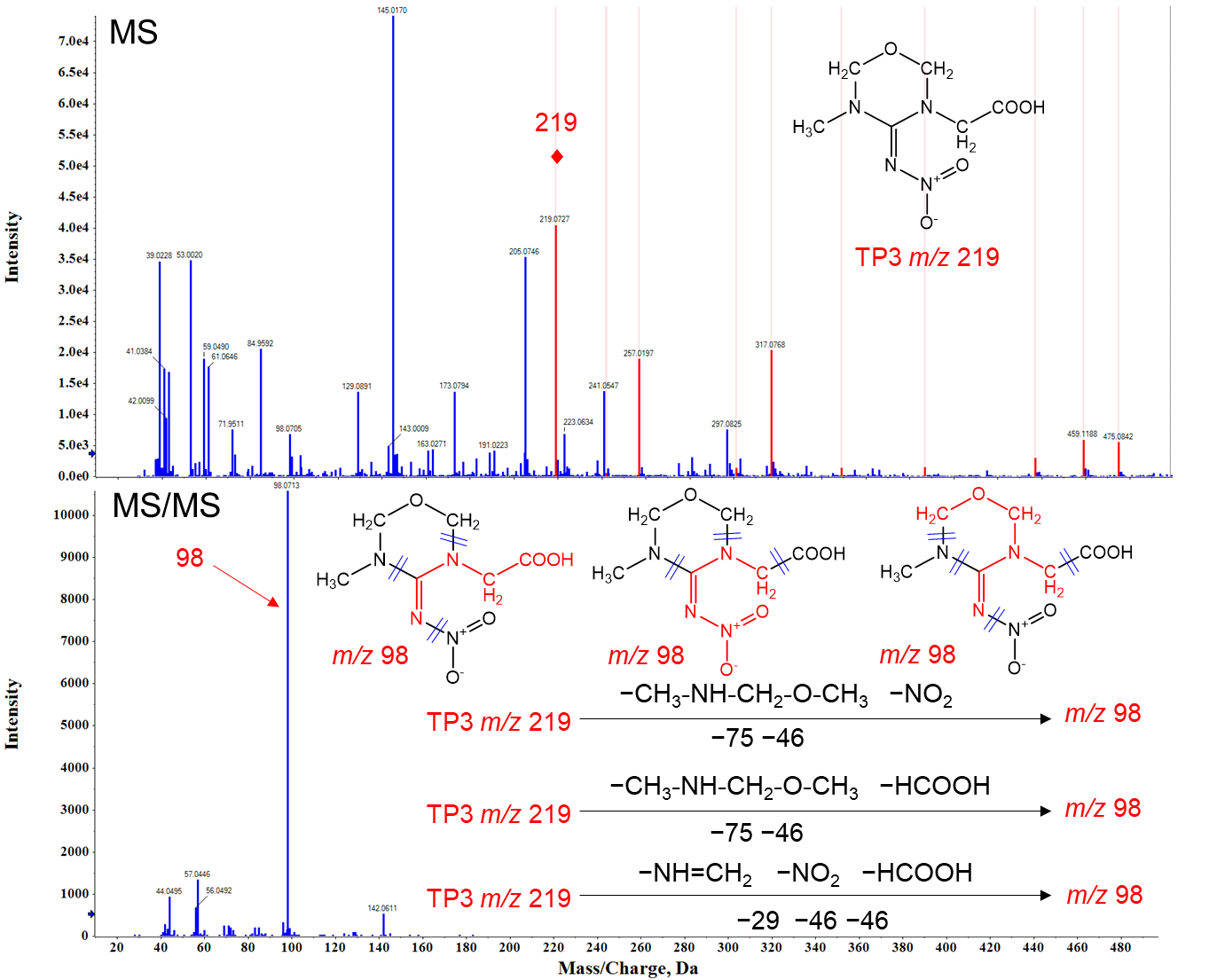


# Fig. S9. QTOF mass spectrums of TP3.


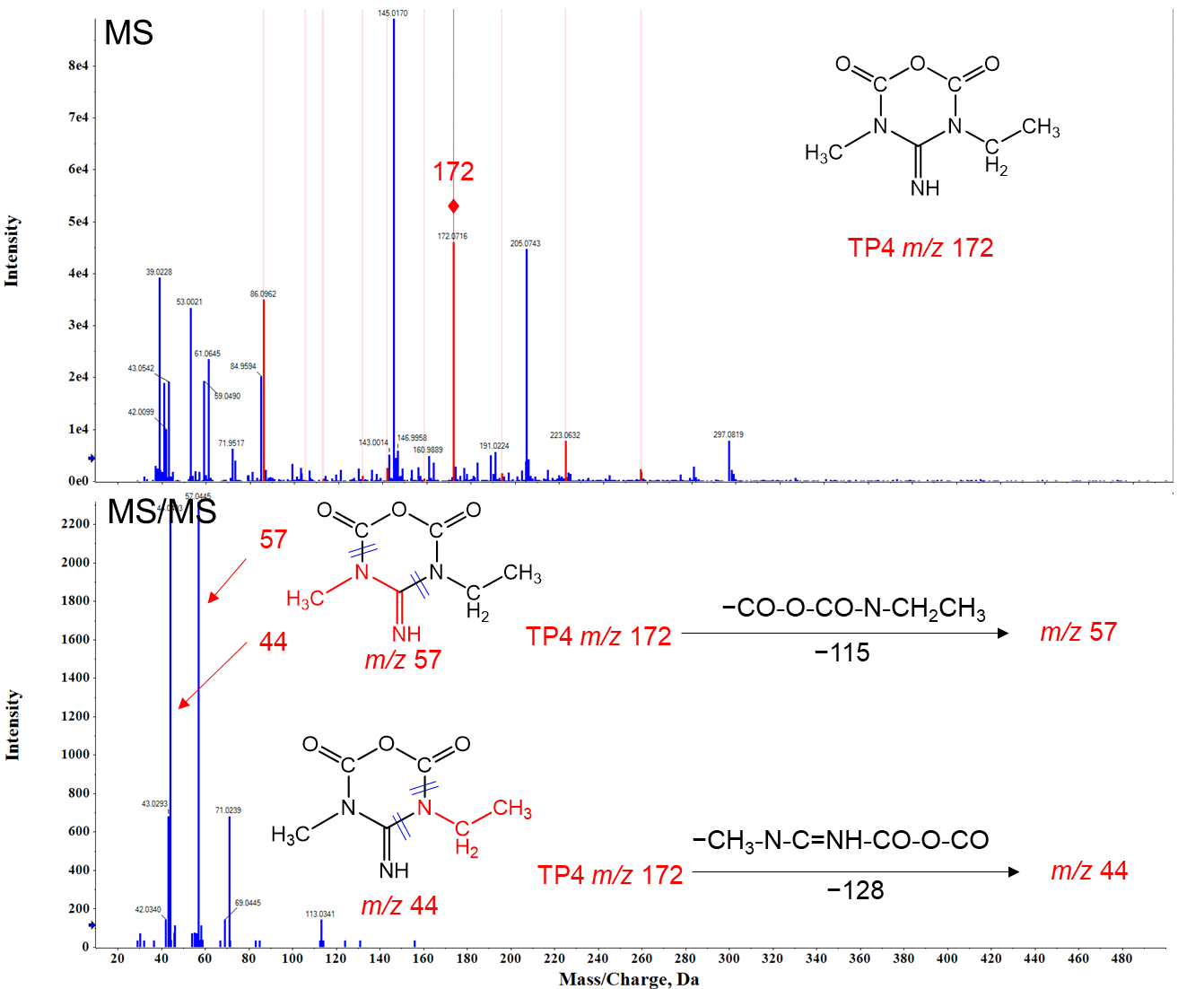


# Fig. S10. QTOF mass spectrums of TP4.


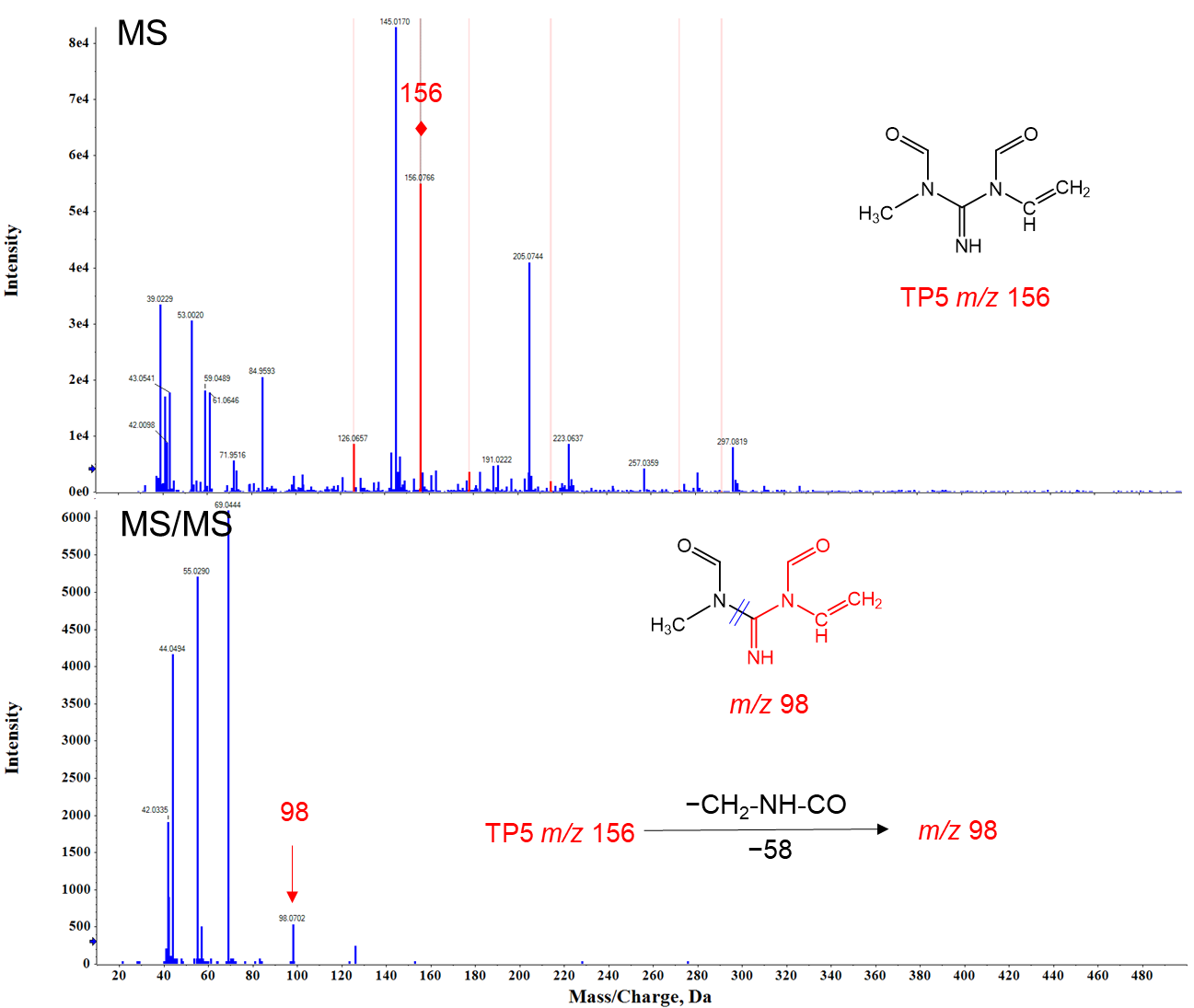


# Fig. S11. QTOF mass spectrums of TP5.


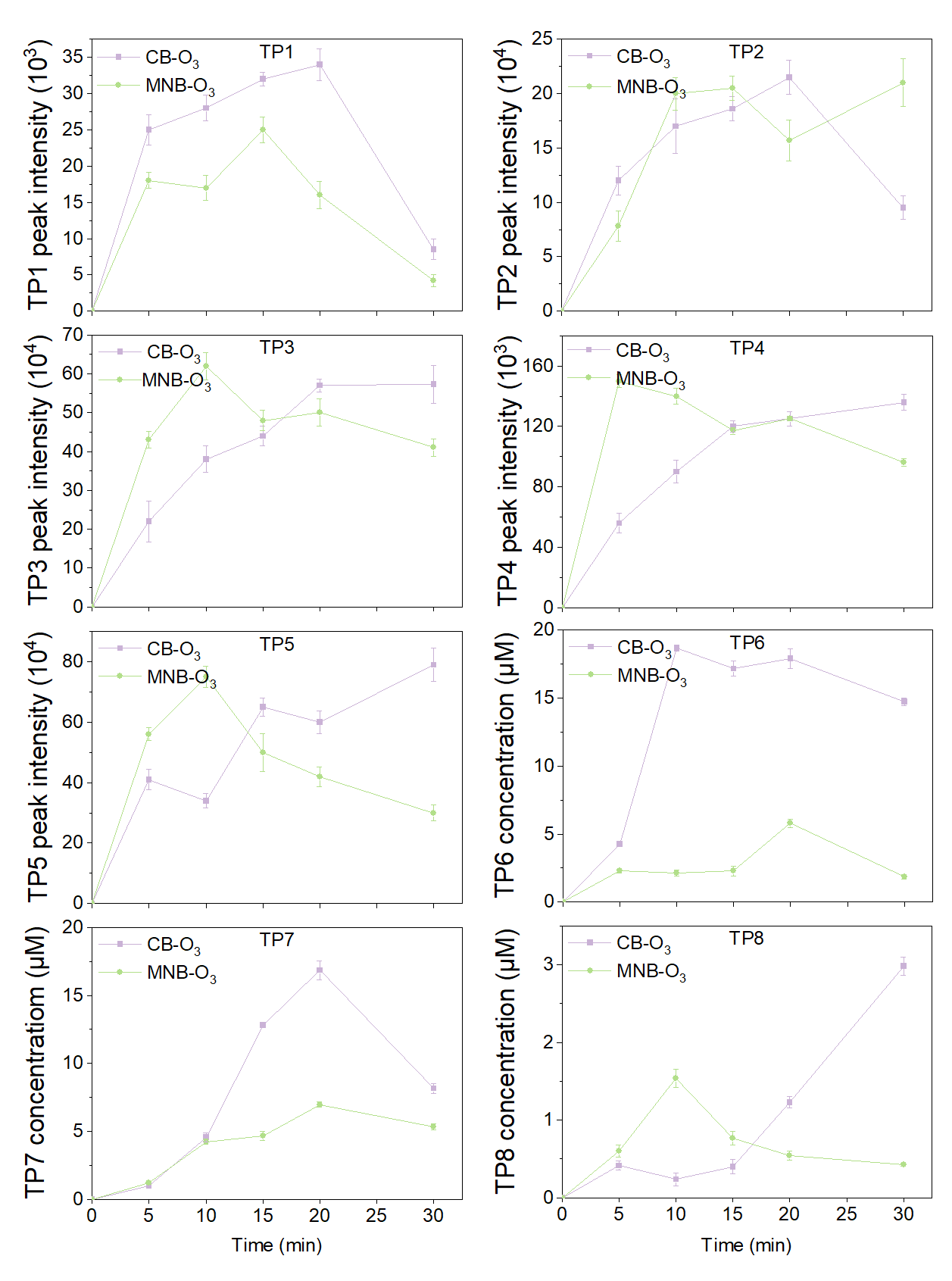


# Fig. S12. The concentrations of various TPs along with the oxidation time.


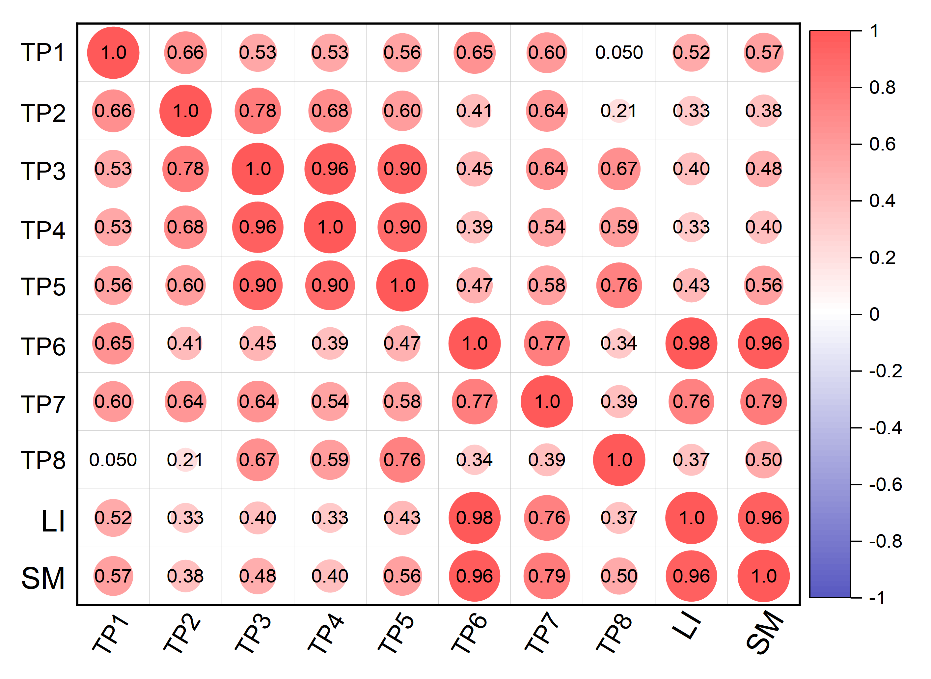


# Fig. S13. The correlation among TP concentrations, luminescent inhibition (LI) of luminescent bacteria and spontaneous movement (SM) of zebrafish.


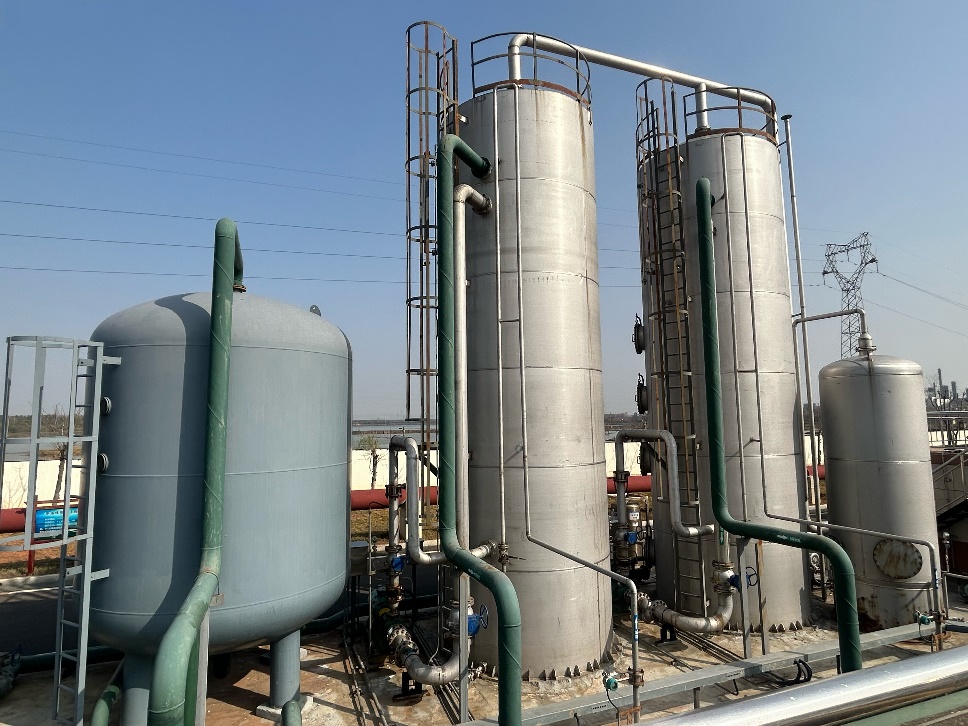


# Fig. S14. Photograph of the MNB-O_3_ treatment towers at the industrial WWTP in Jiangxi Province, China.


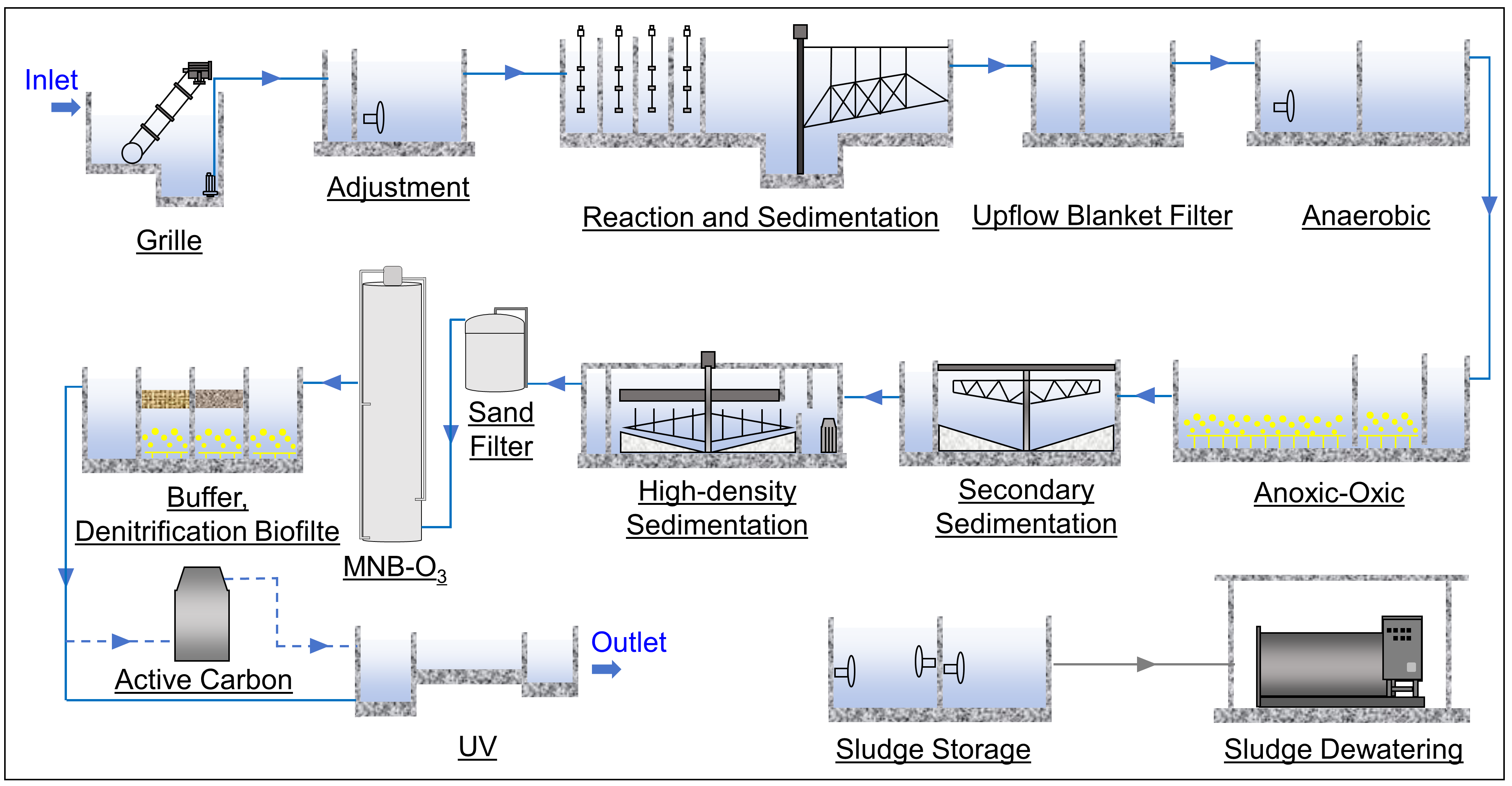


# Fig. S15. The process flow chart of this industrial WWTP.

# Table S1. Water quality of the secondary sedimentation tank effluent from an industrial wastewater treatment plant (mg/L, except for pH).

| **Index** | **pH** | **TOC** | **COD** | **TP** | **TN** | **NH_3_-N** |
| --- | --- | --- | --- | --- | --- | --- |
| value | 7.35 | 20.20 | 51.00 | 0.12 | 7.35 | 0.37 |

# Table S2. Second-order rate constants for the reaction of probes and scavengers used in this study with their related ROS (M^−1^ s^−1^).

| **Probes/Scavenger** | **O_3_** | **^•^OH** | **^1^O_2_** | **O_2_^•−^** |
| --- | --- | --- | --- | --- |
| pCBA | <0.1 [1] | 5E9 [2] | 1.4E7 [3] | 8.6E7 [3] |
| MDE | <1 [3] | 6E9 [3] | 1.5E8 [3] | No reaction [3] |
| MeOH | 0.02 [4] | 9.7E8 [5] | 3E3 [6] | No reaction [3] |
| FFA | 1.6E3 [3] | 1.5E10 [5] | 1.2E8 [6] | 3.5E3 [3] |
| pBQ | 2.5E3 [7] | 1.2E9 [8] | 6.6E7 [3] | 8.3E8 [9] |

# Table S3. The gradient of mobile phase for the instrumental analysis by UPLC-QTOF-MS/MS.

| **Time（min）** | **Mobile phase A**  **0.1% formic acid（%）** | **Mobile phase B**  **MeOH（%）** |
| --- | --- | --- |
| 0.0 | 90 | 10 |
| 8.0 | 10 | 90 |
| 15.0 | 10 | 90 |
| 15.1 | 90 | 10 |
| 20.0 | 90 | 10 |

# Table S4. Hazard classification system

| **Class** | **Ⅰ** | **Ⅱ** | **Ⅲ** | **Ⅳ** | **Ⅴ** |
| --- | --- | --- | --- | --- | --- |
| Toxicity | No acute toxicity | Slight acute toxicity | Acute toxicity | High acute toxicity | Very high acute toxicity |
| TUa | TUa < 0.4 | 0.4 ≤ TUa < 1 | 1 ≤ TUa < 10 | 10 ≤ TUa < 100 | 100 ≤ TUa |

# Table S5. ADCH charge of THM calculated by DFT at the M062X/def2TZVP level.

| **Atom** | **ADCH charge** | **Atom** | **ADCH charge** | **Atom** | **ADCH charge** |
| --- | --- | --- | --- | --- | --- |
| 1(C) | 0.219970 | 11(H) | 0.175326 | 21(C) | 0.212820 |
| 2(C) | −0.000774 | 12(H) | 0.095723 | 22(S) | 0.088635 |
| 3(C) | 0.011426 | 13(N) | −0.392805 | 23(H) | −0.019266 |
| 4(H) | 0.152535 | 14(N) | 0.664689 | 24(C) | 0.047017 |
| 5(H) | 0.128956 | 15(O) | −0.465028 | 25(Cl) | −0.008881 |
| 6(O) | −0.283932 | 16(O) | −0.426129 | 26(N) | −0.314138 |
| 7(N) | −0.055348 | 17(C) | −0.033285 | 27(H) | 0.123376 |
| 8(N) | −0.071716 | 18(H) | 0.123969 | 28(H) | 0.138214 |
| 9(C) | −0.208044 | 19(H) | 0.157807 |  |  |
| 10(H) | 0.094115 | 20(C) | −0.155282 |  |  |

# Table S6. Fukui functions and double descriptors of THM calculated by DFT at the M062X/def2TZVP level.

| **Atom** | **q(N)** | **q(N+1)** | **q(N−1)** | **f^−^** | **f^+^** | **f^0^** | **Δf** |
| --- | --- | --- | --- | --- | --- | --- | --- |
| 1(C) | 0.1597 | 0.1082 | 0.1665 | 0.0068 | 0.0515 | 0.0291 | 0.0447 |
| 2(C) | 0.0796 | 0.0698 | 0.0841 | 0.0044 | 0.0099 | 0.0072 | 0.0054 |
| 3(C) | 0.0897 | 0.0777 | 0.0935 | 0.0038 | 0.012 | 0.0079 | 0.0082 |
| 4(H) | 0.0717 | 0.0628 | 0.0774 | 0.0057 | 0.0089 | 0.0073 | 0.0031 |
| 5(H) | 0.056 | 0.045 | 0.0605 | 0.0045 | 0.011 | 0.0077 | 0.0065 |
| 6(O) | −0.1792 | −0.1897 | −0.1706 | 0.0086 | 0.0106 | 0.0096 | 0.002 |
| 7(N) | −0.0348 | −0.0591 | −0.0262 | 0.0086 | 0.0243 | 0.0165 | 0.0157 |
| 8(N) | −0.0428 | −0.0668 | −0.0291 | 0.0136 | 0.024 | 0.0188 | 0.0104 |
| 9(C) | −0.0262 | −0.039 | −0.0227 | 0.0036 | 0.0127 | 0.0081 | 0.0091 |
| 10(H) | 0.046 | 0.032 | 0.0483 | 0.0024 | 0.014 | 0.0082 | 0.0117 |
| 11(H) | 0.0622 | 0.047 | 0.0658 | 0.0036 | 0.0153 | 0.0094 | 0.0117 |
| 12(H) | 0.0399 | 0.0213 | 0.0436 | 0.0037 | 0.0186 | 0.0112 | 0.0149 |
| 13(N) | −0.1974 | −0.255 | −0.1927 | 0.0046 | 0.0576 | 0.0311 | 0.053 |
| 14(N) | 0.2653 | 0.0939 | 0.2693 | 0.004 | 0.1715 | 0.0877 | 0.1675 |
| 15(O) | −0.3051 | −0.5521 | −0.2956 | 0.0095 | 0.247 | 0.1282 | 0.2375 |
| 16(O) | −0.2829 | −0.5008 | −0.2744 | 0.0085 | 0.2179 | 0.1132 | 0.2094 |
| 17(C) | 0.0141 | 0.0046 | 0.0356 | 0.0215 | 0.0096 | 0.0155 | −0.0119 |
| 18(H) | 0.0575 | 0.0447 | 0.0794 | 0.0219 | 0.0128 | 0.0174 | −0.0091 |
| 19(H) | 0.0671 | 0.0568 | 0.089 | 0.0219 | 0.0103 | 0.0161 | −0.0116 |
| 20(C) | −0.0298 | −0.0304 | 0.1102 | 0.14 | 0.0006 | 0.0703 | −0.1394 |
| 21(C) | −0.0128 | −0.0203 | 0.1566 | 0.1694 | 0.0075 | 0.0884 | −0.1619 |
| 22(S) | 0.1078 | 0.101 | 0.2578 | 0.15 | 0.0068 | 0.0784 | −0.1432 |
| 23(H) | 0.0563 | 0.0479 | 0.1139 | 0.0576 | 0.0083 | 0.033 | −0.0493 |
| 24(C) | 0.0497 | 0.046 | 0.1402 | 0.0905 | 0.0037 | 0.0471 | −0.0868 |
| 25(Cl) | −0.0334 | −0.0388 | 0.1197 | 0.1531 | 0.0054 | 0.0792 | −0.1477 |
| 26(N) | −0.1974 | −0.201 | −0.1293 | 0.0681 | 0.0035 | 0.0358 | −0.0646 |
| 27(H) | 0.0539 | 0.0426 | 0.0599 | 0.006 | 0.0114 | 0.0087 | 0.0054 |
| 28(H) | 0.0652 | 0.0518 | 0.0694 | 0.0042 | 0.0134 | 0.0088 | 0.0092 |

# Table S7. THM and its transformation products identified during ozonation.

| **Compound** | **Formula** | **Structure** | **[M+H] *m/z*** | **Retention time (min)** | **Method** |
| --- | --- | --- | --- | --- | --- |
| THM | C_8_H_10_ClN_5_O_3_S |  | 292.0269, 211.0651, 195.5193, 145.0196, 131.9673 | 5.813 | UPLC-QTOF-MS/MS |
| TP1 | C_6_H_8_ClN_5_O_2_S |  | 250.0156, 242.1542, 145.0173, 131.9663, 113.0163 | 6.703 | UPLC-QTOF-MS/MS |
| TP2 | C_4_H_8_N_4_O_3_ |  | 161.0667, 145.0170, 84.0550, 42.0334 | 3.551 | UPLC-QTOF-MS/MS |
| TP3 | C_6_H_10_N_4_O_5_ |  | 219.0727, 145.0170, 98.0713 | 2.041 | UPLC-QTOF-MS/MS |

Continued Table S6

| **Compound** | **Formula** | **Structure** | **[M+H] *m/z*** | **Retention time (min)** | **Method** |
| --- | --- | --- | --- | --- | --- |
| TP3 | C_6_H_10_N_4_O_5_ |  | 219.0727, 145.0170, 98.0713 | 2.041 | UPLC-QTOF-MS/MS |
| TP4 | C_6_H_9_N_3_O_3_ |  | 172.0716, 145.0170, 86.0962, 57.0445, 44.0493 | 1.875 | UPLC-QTOF-MS/MS |
| TP5 | C_6_H_9_N_3_O_2_ |  | 156.0766, 145.0170, 126.0657, 98.0702 | 1.775 | UPLC-QTOF-MS/MS |
| TP6 | CH_2_O |  | / | 5.36 | UPLC-UV |
| TP7 | C_2_H_4_O |  | / | 7.502 | UPLC-UV |
| TP8 | C_3_H_4_O |  | / | 10.9 | UPLC-UV |

# Table S8. Toxicity of THM and its transformation products by ozonation (data from ECOSAR and T.E.S.T. calculations).

| **Chemical** | **Acute toxicity (mg/L)** | | | | **Developmental toxicity** | | **mutagenicity** | **Bioaccumulation factor** |
| --- | --- | --- | --- | --- | --- | --- | --- | --- |
|  | **fish**  **LC_50_ (96 hr)** | **water flea**  **LC_50_ (48 hr)** | **algae**  **LC_50_ (48 hr)** | |  |  |  |  |
| THM | 346.00 | 35.80 | 39.00 | | 1.04 | | 1.00 | 7.74 |
| TP1 | 373.00 | 37.90 | 42.80 | | 0.6 | | 1.00 | 2.44 |
| TP2 | 2.02E3 | 175.00 | 272.00 | | 0.52 | | 1.00 | 0.79 |
| TP3 | 1.32E6 | 8.47E4 | 2.38E5 | | 0.85 | | 0.86 | 0.24 |
| TP4 | 1.00E3 | 857.00 | 0.54 | | **1.25** | | 0.36 | 1.38 |
| TP5 | 527.00 | 632.00 | 38.50 | | **1.10** | | 0.55 | 0.36 |
| TP6 | **11.20** | **12.00** | **5.87** | | / | | 0.36 | 0.88 |
| TP7 | **29.00** | **32.60** | **15.10** | | 0.49 | | 0.11 | 2.5 |
| TP8 | **0.12** | 969.00 | 273.00 | | 0.64 | | 0.51 | 3.41 |
| Note |  | very toxic / positive | |  | | toxic | |  |
|  |  | harmful | |  | | not harmful or negative | |  |

# Table S9. Water quality of the wastewater before and after treatment with MNB-O_3_ in large-scale application (mg/L, except for pH).

| **Index** | **pH** | **TOC** | **COD** | **TP** | **TN** | **NH_3_-N** |
| --- | --- | --- | --- | --- | --- | --- |
| Before | 7.19 ± 0.23 | 20.20± 3.54 | 63.05± 10.74 | 0.47± 0.18 | 13.21± 1.59 | 0.61± 0.25 |
| After | 6.21± 0.35 | 13.54± 2.88 | 41.50± 5.24 | 0.42± 0.05 | 5.31± 0.73 | 0.25± 0.07 |

# Table S10. Power of electrical equipment involved in the large-scale application.

| **Equipment** | **Set** | **Power (kW)** |
| --- | --- | --- |
| Ozone generator | 1 | 25 |
| internal circulation pump | 2 | 1.1 |
| external circulation pump | 2 | 3 |
| ozone tower circulation pump | 2 | 30 |
| induced draft fan | 2 | 2.2 |
| tail gas treatment device | 1 | 6.7 |

# References:

[1] Yao, C.C.D. and Haag, W.R., Rate constants for direct reactions of ozone with several drinking water contaminants, Water Research 25 (1991) 761–773. 10.1016/0043-1354(91)90155-J

[2] Wu, C., De Visscher, A. and Gates, I.D., Reactions of hydroxyl radicals with benzoic acid and benzoate, RSC Advances 7 (2017) 35776–35785. 10.1039/c7ra05488b

[3] Guo, Y., Long, J., Huang, J., Yu, G. and Wang, Y., Can the commonly used quenching method really evaluate the role of reactive oxygen species in pollutant abatement during catalytic ozonation?, Water Research 215 (2022) 118275. 10.1016/j.watres.2022.118275

[4] Hoigné, J. and Bader, H., Rate constants of reactions of ozone with organic and inorganic compounds in water—I: Non-dissociating organic compounds, Water Research 17 (1983) 173–183. 10.1016/0043-1354(83)90098-2

[5] Buxton, G.V., Greenstock, C.L., Helman, W.P. and Ross, A.B., Critical review of rate constants for reactions of hydrated electrons, hydrogen atoms and hydroxyl radicals (·OH/·O^−^) in aqueous solution, Journal of Physical and Chemical Reference Data 17 (1988) 513–886. 10.1063/1.555805

[6] Wilkinson, F., Helman, W.P. and Ross, A.B., Rate constants for the decay and reactions of the lowest electronically excited singlet state of molecular oxygen in solution. An expanded and revised compilation, Journal of Physical and Chemical Reference Data 24 (1995) 663–677. 10.1063/1.555965

[7] Mvula, E. and Von Sonntag, C., Ozonolysis of phenols in aqueous solution, Organic and Biomolecular Chemistry 1 (2003) 1749–1756. 10.1039/B301824P

[8] G.E., A. and B.D., M., Pulse radiolysis of benzoquinone and hydroquinone. Semiquinone formation by water elimination from trihydroxy-cyclohexadienyl radicals, Transactions of the Faraday Society 63 (1967) 1171–1180. 10.1039/TF9676301171

[9] Guo, Y., Zhang, Y., Yu, G. and Wang, Y., Revisiting the role of reactive oxygen species for pollutant abatement during catalytic ozonation: The probe approach versus the scavenger approach, Applied Catalysis B: Environmental 280 (2021) 10.1016/j.apcatb.2020.119418
